# An Enhanced Human Evolutionary Optimization Algorithm for Global Optimization and Multi-Threshold Image Segmentation

**DOI:** 10.3390/biomimetics10050282

**Published:** 2025-05-01

**Authors:** Liang Xiang, Xiajie Zhao, Jianfeng Wang, Bin Wang

**Affiliations:** 1 Department of Space and Culture Design Graduate School of Techno Design (TED), Kookmin University, Seoul 02707, Republic of Korea; xiangliang68@kookmin.ac.kr; 2 College of Design, Hanyang University, Ansan 15588, Republic of Korea; zhaoxiajie@hanyang.ac.kr (X.Z.); wangbin19045@hanyang.ac.kr (B.W.)

**Keywords:** human evolutionary optimization algorithm, CEC 2017, image segmentation, learning strategy, optimization algorithm, 65K05

## Abstract

Thresholding image segmentation aims to divide an image into a number of regions with different feature attributes in order to facilitate the extraction of image features in the context of image detection and pattern recognition. However, existing threshold image-segmentation methods suffer from the problem of easily falling into locally optimal thresholds, resulting in poor image segmentation. In order to improve the image-segmentation performance, this study proposes an enhanced Human Evolutionary Optimization Algorithm (HEOA), known as CLNBHEOA, which incorporates Otsu’s method as an objective function to significantly improve the image-segmentation performance. In the CLNBHEOA, firstly, population diversity is enhanced using the Chebyshev–Tent chaotic mapping refraction opposites-based learning strategy. Secondly, an adaptive learning strategy is proposed which combines differential learning and adaptive factors to improve the ability of the algorithm to jump out of the locally optimum threshold. In addition, a nonlinear control factor is proposed to better balance the global exploration phase and the local exploitation phase of the algorithm. Finally, a three-point guidance strategy based on Bernstein polynomials is proposed which enhances the local exploitation ability of the algorithm and effectively improves the efficiency of optimal threshold search. Subsequently, the optimization performance of the CLNBHEOA was evaluated on the CEC2017 benchmark functions. Experiments demonstrated that the CLNBHEOA outperformed the comparison algorithms by over 90%, exhibiting higher optimization performance and search efficiency. Finally, the CLNBHEOA was applied to solve six multi-threshold image-segmentation problems. The experimental results indicated that the CLNBHEOA achieved a winning rate of over 95% in terms of fitness function value, peak signal-to-noise ratio (PSNR), structural similarity (SSIM) and feature similarity (FSIM), suggesting that it can be considered a promising approach for multi-threshold image segmentation.

## 1. Introduction

Image segmentation aims to divide an image into several regions with different feature attributes in order to facilitate the extraction of important feature information, which is a step preliminary to image detection and pattern recognition. Currently, image-segmentation techniques are widely used in remote sensing satellites [1], medical images [2], intelligent transportation [3] and space technology [4]. Common image-segmentation methods mainly include threshold segmentation methods [5], clustering segmentation methods [6] and region extraction methods [7]. The high processing efficiency and high reliability performance of the threshold segmentation method make it one of the most widely used image-segmentation techniques [8]. The most critical factor of the threshold segmentation method lies in the identification of the optimal segmentation threshold, and the current commonly employed method is to model it as an optimization problem, maximize the inter-class variance of different regions as the objective function using Otsu’s method [9], and search for the optimal segmentation threshold using meta-heuristic optimization algorithms, which makes it possible to minimize the cost of the search while determining the optimal threshold [10].

Currently, there are four main types of common metaheuristic optimization algorithms, namely, evolutionary algorithms, swarm intelligence algorithms, physical and chemical algorithms and human-based algorithms [11]. Among the main representatives of evolutionary algorithms are Genetic Algorithm (GA) [12], Differential Evolution (DE) [13] and Evolutionary Strategies (ES) [14]. Some typical representatives of swarm intelligence algorithms are Particle Swarm Optimization (PSO) [15], Ant Colony Optimization (ACO) [16] and Gray Wolf Optimizer (GWO) [17]. Some typical representatives of physical and chemical algorithms are Multi-Verse Optimizer (MVO) [18], Magnetic Optimization Algorithm (MOA) [19] and Atom Search Optimization (ASO) [20]. The main representatives of human-based algorithms are Teaching–Learning Based Optimization (TLBO) [21], Search and Rescue Optimization (SAR) [22] and Student Psychology-Based Optimization (SPBO) [23]. Thanks to the lightweight computational characteristics of the meta-heuristic optimization algorithm, researchers have widely combined it with the Otsu method to propose image-segmentation methods based on the meta-heuristic optimization algorithm.

For example, to address the low real-time efficiency of existing Otsu methods, Huang et al. [24] proposed an image-segmentation method (FOA-OTSU) that combines the Fruit Fly Optimization Algorithm (FOA) and the Otsu algorithm with the aim of improving the real-time efficiency of Otsu segmentation. The experimental results show that this method significantly reduces the segmentation time while keeping the segmentation effect unchanged, exhibiting faster convergence and higher real-time performance. Ma et al. [25] proposed an improved Whale Optimization Algorithm (RAV-WOA) for multi-threshold image segmentation using the Otsu method as the objective function. The global search and local exploitation capabilities of the algorithm are improved by introducing a reverse learning strategy and an adaptive weighting strategy. The experimental results confirm that the proposed RAV-WOA outperforms other algorithms in terms of convergence speed and accuracy, as well as segmentation quality and stability, and is suitable for the segmentation tasks associated with grayscale and color images. Chen et al. [26] proposed a new adaptive fractional order genetic particle swarm optimization (FOGPSO) algorithm for improving the search performance of the Otsu image-segmentation algorithm. The algorithm combines the advantages of genetic algorithm and particle swarm optimization by adaptively adjusting the fractional-order calculus operators to optimize the velocity and position updates of the particles. The experimental results show that FOGPSO outperforms other existing methods in several metrics, such as region contrast and peak signal-to-noise ratio, proving its effectiveness and superiority in image segmentation. Qin et al. [27] proposed an Otsu multi-threshold segmentation algorithm based on an improved ant colony optimization algorithm. By combining the Levy flight pattern and global transfer probability in the search process of the algorithm, a faster convergence speed and a more effective threshold search are realized. The experimental results show that the algorithm outperforms the traditional Otsu algorithm, as well as the Otsu algorithm based on the traditional ant colony optimization algorithm, in finding the optimal thresholds. Fan et al. [28] proposed an Otsu image-segmentation algorithm based on fractional order Moth–Flame Optimization (MFO), aiming to solve the problems of low segmentation accuracy, slow convergence and tendency to fall into local optimality, which the traditional MFO algorithm is associated with in image-segmentation processing. The position of the Moth–Flame population is adaptively adjusted by utilizing the memory and heritability of fractional order differentiation. The experimental results show that the algorithm outperforms the traditional MFO algorithm in terms of convergence speed, segmentation accuracy and fitness function value. Abdul et al. [29] proposed a multilevel threshold segmentation method based on Gray Wolf Optimizer (GWO) to solve the problem of the excessive computational complexity of traditional methods when the number of thresholds increases. Its performance has been verified by using standard test images and comparisons with PSO and BFO methods, and the results show that the proposed method has significant advantages in terms of stability, segmentation quality and computational speed. Wu et al. [30] proposed an improved Teaching–Learning-Based Optimization algorithm (DI-TLBO) and applied it to the multi-threshold image-segmentation problem. By introducing random numbers, self-feedback learning and variance crossover strategies, DI-TLBO is made to perform well in global optimization and exploration ability. The experimental results show that the DI-TLBO-based method has high accuracy and stability in segmentation of standard test images and cast X-ray images and can effectively recognize and segment defects in images. Mohamed et al. [31] proposed an improved WHALE optimization algorithm (MLWOA) for multilevel threshold image segmentation. By integrating memory mechanism, multi-leader approach, self-learning strategy and the Levy flight method, MLWOA avoids the limitations of traditional WOA. The experimental results show that MLWOA performs well in terms of image-segmentation performance metrics when using Otsu method as the fitness function.

The above study combines the optimization algorithm with the Otsu method to propose an image-segmentation method based on the optimization algorithm, which greatly enhances the image-segmentation performance of the Otsu method and helps to better extract the image feature information. But although the current optimization algorithms have achieved good results in the field of image segmentation, with the increase of segmentation dimension, the existing methods still have the problem of easily falling into the locally optimal segmentation threshold, which results in poor image-segmentation results. Therefore, there is a need to explore a novel, efficient and robust optimization tool to better cope with the above problems. Fortunately, the Human Evolutionary Optimization Algorithm (HEOA) [32] has been shown to be a robust method with efficient optimization performance, and studies have also demonstrated that it is highly scalable; additionally, it is not currently utilized in image segmentation. Therefore, in order to alleviate the limitations of the existing threshold segmentation methods, and at the same time expand the application areas of HEOA, this study applies HEOA to solve the multi-threshold image-segmentation problem. In addition, considering the challenges associated with the increase in the number of thresholds for image segmentation and in order to further utilize the efficient performance of HEOA, this study proposes an enhanced HEOA, one based on HEOA and combining four learning strategies, which is referred to as CLNBHEOA. In this algorithm, firstly, the Chebyshev–Tent chaotic mapping refraction opposites-based learning strategy is used to ensure that the initial population is associated with a better solution space traversal ability. Secondly, an adaptive learning strategy is proposed, an approach which improves the ability of the algorithm to jump out of the locally optimal threshold by learning from individual information gaps possessing different properties while incorporating adaptive factors. In addition, a nonlinear control factor is proposed to better balance the global exploration phase and the local exploitation phase of the algorithm. Finally, a three-point guidance strategy based on Bernstein polynomials is proposed to enhance the local exploitation of the algorithm. The main contributions of this paper are as follows:

- The initialization population scheme based on Chebyshev–Tent chaotic mapping refraction opposites-based learning strategy is proposed to enhance the global search capability of the algorithm.
- An adaptive learning strategy is proposed which improves the ability of the algorithm to jump out of the locally optimum threshold by learning from the information gaps of individuals possessing different properties, while incorporating adaptive factors.
- Nonlinear control factor is proposed to better balance the global exploration phase and the local exploitation phase of the algorithm to improve the image threshold segmentation performance.
- A three-point guidance strategy based on Bernstein polynomials is proposed to enhance the local exploitation of the algorithm.
- Combining the above four learning strategies, the CLNBHEOA is proposed and applied to solve the multi-threshold image-segmentation problem to achieve better image-segmentation performance.

The subsequent portions of the work are structured as follows: Section 2 outlines the mathematical model and execution logic of HEOA. Section 3 provides a detailed overview of the four learning strategies proposed in this paper and proposes the CLNBHEOA in conjunction with the learning strategies. Section 4 evaluates the optimization performance of the CLNBHEOA using the CEC2017 function test set [33]; the results confirm that the CLNBHEOA is a method that possesses efficient optimization capabilities. In Section 5, image-segmentation experiments are conducted on six images using the CLNBHEOA, and the experimental results show that it has efficient image-segmentation performance. Section 6 gives the conclusion of this paper and future research directions.

## 2. Related Works

Inspired by human evolutionary behaviors in complex environments, including human adaptation to the environment as well as the search for better individuals, the Human Evolutionary Optimization Algorithm (HEOA) was proposed as a novel meta-heuristic algorithm in 2024; it consists of three main phases: the population initialization phase, the human exploration phase and the human development phase. These will be mathematically modeled in detail in the following.

### 2.1. Population Initialization Phase

At the initial stage of human evolution, the purpose of population initialization using the logical chaotic mapping method is to generate a set of initialized individuals that will serve as the initial solution set for the optimization problem, in which each individual represents a candidate solution to the optimization problem, The individuals are generated by Equation (1). The N individuals are subsequently pooled into an initialized population $pop=[X_{1},X_{2},\ldots ,X_{i},\ldots ,X_{N}]$.

(1) $$X_{i}^{1}=LB+(UB-LB)\cdot L_{i},\;1\leq i\leq N$$

where $X_{i}^{1}$ denotes the ith individual in the initial population; UB and LB denote the upper and lower boundary constraints of the problem to be optimized, respectively; the vector of size 1×Dim, Dim denotes the dimensionality of the variables of the problem to be optimized; N denotes the size of the population; and $L_{i}$ denotes the ith component of the sequence of logical chaotic mapping, computed using Equation (2):

(2) $$L_{i}=\alpha \cdot L_{i-1}\cdot (1-L_{i-1}),\;0\leq L_{0}\leq 1,\;i=1,2,\ldots ,N,\;\alpha =4$$

### 2.2. Human Exploration Phase

After generating the initial population, individuals improve the algorithm’s optimization accuracy by exploring unknown regions in order to enable the algorithm to explore regions in which the existence of a globally optimal solution is more promising. Specifically, in the human exploration phase, individual information is computed through Equation (3):

(3) $$\begin{array}{cc} X_{i}^{t+1} & =\beta \cdot \left(1-\frac{t}{Max_{iter}}\right)\cdot (X_{i}^{t}-X_{best})\cdot Levy(dim)+\\ & X_{best}\cdot \left(1-\frac{t}{Max_{iter}}\right)+(X_{mean}^{t}-X_{best})\cdot floor\left(\frac{rand}{f_{jump}}\right)\cdot f_{jump} \end{array}$$

where t denotes the current iteration number, $Max_{iter}$ denotes the maximum number of iterations of the algorithm, $X_{i}^{t+1}$ denotes the information of the ith individual in the (t+1)th iteration, $X_{i}^{t}$ denotes the information of the ith individual in the tth iteration, $X_{best}$ denotes the optimal individual in the current population, floor(·) denotes a downward rounding operation, rand denotes the pseudo-random number generated in the interval [0, 1], and $X_{mean}^{t}$ denotes the average information of all the individuals in the current population, which is computed by using Equation (4):

(4) $$X_{mean}^{t}=\frac{1}{N}\sum_{i=1}^{N}X_{i}^{t}$$

β denotes the adaptive function, which is computed using Equation (5):

(5) $$\beta =0.2\cdot \left(1-\frac{t}{Max_{iter}}\right)\cdot \left(X_{i}^{t}-X_{mean}^{t}\right)$$

Levy(dim) denotes the Levy distributed random number with parameter dim, computed using Equation (6):

(6) $$\left\{\begin{array}{l} Levy(dim)=\frac{\mu \cdot \sigma }{|\nu {|}^{\gamma }}\\ \mu \;\sim N(0,dim)\\ \nu \;\sim N(0,dim)\\ \sigma ={\left(\frac{\Gamma (1+\gamma )\cdot \sin\left(\frac{\pi \gamma }{2}\right)}{\Gamma \left(\frac{1+\gamma }{2}\right)\cdot \gamma \cdot 2^{\frac{1+\gamma }{2}}}\right)}^{\gamma +1} \end{array}\right.$$

where γ takes the value of 1.5 and sin(·) denotes the sinusoidal computational function. Finally, $f_{jump}$ denotes the jump coefficient, computed using Equation (7):

(7) $$f_{jump}=\frac{\left(LB(1)-UB(1)\right)}{\delta },\;\delta \in [100,2000]$$

### 2.3. Human Development Phase

As mentioned earlier, the human exploration phase locates the potential globally optimal solution region, and then the human development phase requires further exploitation of the potentially optimal region to improve the algorithm’s convergence speed and optimization accuracy. Specifically, the human development phase consists of four roles, i.e., leaders, explorers, followers and losers, each of which employs a different search strategy to make the global optimal solution easier to locate by utilizing diversified search strategies. The following section describes in detail the individual update strategies corresponding to the four roles.

#### 2.3.1. Leaders Update Strategy

Define the top 40% of individuals in the population as associated with the smallest value of the fitness function as leaders; leaders need to further develop their surrounding area to further improve the optimization accuracy of the algorithm; since they already have a large amount of knowledge, the update strategy of leaders is expressed using Equation (8).

(8) $$X_{i}^{t+1}=\left\{\begin{array}{ll} \omega \cdot X_{i}^{t}\cdot \exp\left(\frac{-t}{rand\cdot Max_{iter}}\right), & R<A\\ \omega \cdot X_{i}^{t}+Rn\cdot ones(1,dim), & R\geq A \end{array}\right.$$

where exp(·) denotes the exponential operation with constant e as the base, R denotes the pseudo-random number generated in the interval [0, 1], A is set to the constant 0.6, Rn denotes the random number obeying the standard normal distribution, ones(1,dim) denotes the generation of the all-ones vector with the size of 1×dim and ω denotes the knowledge acquisition ease coefficient, which is computed by using Equation (9).

(9) $$\omega =0.2\cdot \cos\left(\frac{\pi }{2}\cdot \left(1-\frac{t}{Max_{iter}}\right)\right)$$

where cos(·) denotes the cosine calculation function.

#### 2.3.2. Explorers Update Strategy

Individuals in the population that rank between 40% and 80% in terms of their fitness function values are defined as explorers; these are designed to explore unknown areas, giving the algorithm an advantage in discovering the globally optimal solution. The updating strategy for the explorers is expressed using Equation (10).

(10) $$X_{i}^{t+1}=Rn\cdot \exp\left(\frac{{\left(X_{worst}^{t}\right)}^{2}-{\left(X_{i}^{t}\right)}^{2}}{i^{2}}\right)$$

where $X_{worst}^{t}$ denotes the individual with the worst fitness function value in the tth iteration.

#### 2.3.3. Followers Update Strategy

Individuals that rank between 80% and 90% in terms of their fitness function values within the population are defined as followers; the follower information is guided by the current optimal individuals in order to rapidly improve the quality of the individuals; specifically, the updating strategy for the followers is expressed using Equation (11).

(11) $$X_{i}^{t+1}=X_{i}^{t}+\omega \cdot Rd\cdot (X_{best}^{t}-X_{i}^{t})$$

where $X_{best}^{t}$ denotes the optimal individual in the tth iteration and Rd denotes a random number in the interval [1, dim].

#### 2.3.4. Losers Update Strategy

Define the last 10% of individuals in the population in terms of the fitness function value as losers, since the quality of losers’ individuals is very low. In order to significantly improve the quality of this part of the individuals, the globally optimal individuals are used for substitution as well as bootstrapping; the losers’ updating strategy is expressed using Equation (12).

(12) $$X_{i}^{t+1}=X_{best}+(X_{best}-X_{i}^{t})\cdot Rn$$

### 2.4. Implementation of HEOA

In this section, the execution logic of HEOA is basically introduced. When using HEOA to solve the optimization problem, first, the initialization population operation is carried out, and it then enters the main loop of the algorithm, and the individual information is updated through the combination of the human exploration phase and the human development phase, so that the quality of the candidate solution is effectively improved. Finally, when the number of loops reaches the maximum number of algorithmic iterations, it outputs the best individual information, which is the best solution to the optimization problem. In order to describe this execution logic in detail, Algorithm 1 gives the execution pseudo-code of HEOA.
**Algorithm 1:** The pseudo-code of the HEOA
  **Input:** N, Dim, UB, LB, $Max_{iter}$, A=0.6
  **Output:** global best solution $X_{best}$ %Population initialization phase Generate initialized populations $pop=[X_{1},X_{2},\ldots ,X_{i},\ldots ,X_{N}]$ according to Equation (1) Calculate the fitness function value for the population $fit=[fit_{1},fit_{2},\ldots ,fit_{i},\ldots ,fit_{N}]$ *for* $t=1:Max_{iter}$ [~, ind] = sort(fit) $X_{best}=pop(ind(1))$ *for* i=1:N    *if* $t\leq (1/4)\cdot Max_{iter}$       %Human exploration phase       Update individual information according to Equation (3)    *else*       %Human development phase       Categorized individuals into four roles based on the fitness function values fit: Leaders, Explores,       Followers, Losers       *if* individuals belong leaders         %Leaders update strategy         Update individual information according to Equation (8)       *end*       *if* individuals belong explores         %Leaders update strategy         Update individual information according to Equation (10)       *end*       *if* individuals belong followers         %Followers update strategy         Update individual information according to Equation (11)       *end*       *if* individuals belong losers         %Followers update strategy         Update individual information according to Equation (12)       *end*    *end* *end* *end* Output global best solution $X_{best}$

## 3. Proposed Model

The original HEOA has the problem of falling into the locally optimal thresholds, resulting in poor image segmentation when dealing with the multi-threshold image-segmentation problem. The essential reason for this is that HEOA has a lack of population diversity in its solution process, which leads to the loss of the algorithm’s global search ability and the ability to jump out of the locally optimal threshold; at the same time, after locating the potentially optimal threshold region, HEOA’s local exploitation ability is also somewhat insufficient. In addition, the HEOA algorithm does not fully consider the balance between the global exploration phase and the local exploitation phase; the above algorithmic defects lead to the losses in the convergence speed and optimization accuracy of the algorithm. To alleviate the above problem, an enhanced HEOA algorithm, called the CLNBHEOA, is proposed in this section, combining four learning strategies. Firstly, the Chebyshev–Tent chaotic mapping refraction opposites-based learning strategy based on Chebyshev–Tent chaotic mapping is proposed for use as an initialization population scheme, which enables the initial population to have a better solution space traversal and improves the global search capability of the algorithm. Secondly, the adaptive learning strategy is proposed to improve the algorithm’s ability to jump out of the locally optimum threshold by learning from individual information gaps possessing different properties, while incorporating adaptive factors. In addition, a nonlinear control factor is proposed to better balance the global exploration phase and the local exploitation phase of the algorithm. Finally, a three-point guidance strategy based on Bernstein polynomials is proposed to enhance the local exploitation of the algorithm. Through the above improvements to the algorithm, the optimization accuracy of the algorithm in solving the multi-threshold image-segmentation problem is effectively improved, and the performance of the algorithm is enhanced. The above improvement points are described in detail in the following, wherein the CLNBHEOA in this study is proposed.

### 3.1. Chebyshev–Tent Chaotic Mapping Refraction Opposites-Based Learning Strategy

Li et al. [34] pointed out that by introducing a chaotic mapping strategy for population initialization, the algorithm can effectively enhance the traversal and non-repeatability of the algorithm throughout the iterative process and improve the algorithm population diversity. Meanwhile, Yang et al. [35] pointed out that opposition learning can effectively enhance the algorithm search diversity and efficiency by generating opposing candidate solutions. Based on this inspiration, this section proposes a population initialization scheme for a Chebyshev–Tent chaotic mapping refraction opposites-based learning strategy to enhance the quality of initial candidate solutions; this in turn improves the algorithm’s global search capability. The specific implementation of the initialization strategy is introduced in detail below. Firstly, the Chebyshev chaotic mapping sequence is computed using Equation (13), and the Tent chaotic mapping sequence is computed using Equation (14).

(13) $$C_{i+1}=\cos\;(\phi \cdot \cos^{-1}\cdot C_{i})$$

where i denotes the number of mappings, $C_{i}$ denotes the function mapping value for the ith time of Chebyshev mapping, ϕ denotes the control parameter, which takes the value of a constant 5, and $C_{0}$ is set to the constant 0.152.

(14) $$t_{i+1}=\left\{\begin{array}{l} \frac{t_{i}}{u},0\leq t_{i}\leq u\\ \frac{1-t_{i}}{1-u},u<t_{i}\leq 1 \end{array}\right.$$

where i denotes the number of mappings, $t_{i}$ denotes the mapped value of the function for the ith time of Tent mapping, u denotes the control parameter, which takes the value of constant 0.4, and $t_{0}$ is set to the constant 0.152.

Subsequently, the Chebyshev population $pop_{c}=[X_{1}^{c},X_{2}^{c},\ldots ,X_{i}^{c},\ldots ,X_{N}^{c}]$ was generated using the Chebyshev chaotic mapping sequence generated above; similarly, the Tent population $pop_{T}=[X_{1}^{T},X_{2}^{T},\ldots ,X_{i}^{T},\ldots ,X_{N}^{T}]$ was generated using the Tent chaotic mapping sequence, with the information of individual individuals being calculated using Equations (15) and (16), respectively.

(15) $$X_{i}^{c}=LB+(UB-LB)\cdot C_{i},\;1\leq i\leq N$$

(16) $$X_{i}^{T}=LB+(UB-LB)\cdot t_{i},\;1\leq i\leq N$$

**Figure 1:**
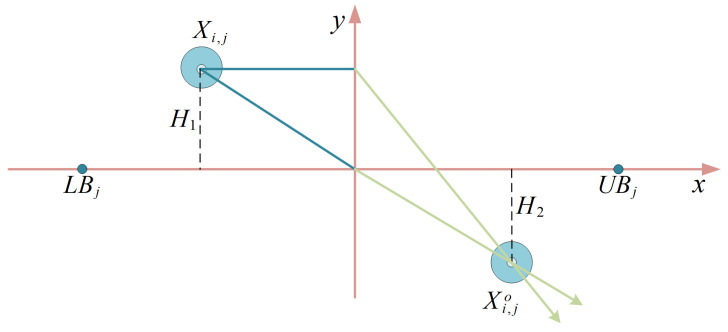
The refraction opposites-based learning strategy simulation diagram.

Subsequently, the refractive opposition learning strategy was utilized to generate corresponding Chebyshev refractive opposition population $pop_{c}^{o}$ and Tent refractive opposition population $pop_{T}^{o}$ based on Chebyshev population and Tent population. Single individuals were calculated using Equations (17) and (18), respectively.

(17) $$X_{i,j}^{c,o}=\frac{UB_{j}+LB_{j}}{2}+\frac{UB_{j}+LB_{j}}{2\delta }-\frac{X_{i,j}^{c}}{\delta }$$

(18) $$X_{i,j}^{T,o}=\frac{UB_{j}+LB_{j}}{2}+\frac{UB_{j}+LB_{j}}{2\delta }-\frac{X_{i,j}^{T}}{\delta }$$

where $X_{i,j}^{c,o}$ denotes the jth dimension information of the ith Chebyshev individual refracting the opposing individual and $X_{i,j}^{T,o}$ denotes the jth dimension information of the ith Tent individual refracting the opposing individual. The term δ is defined as the scaling factor $H_{1}/H_{2}=1.5$ and the values of $H_{1}$ and $H_{2}$ are referenced to the refracting opposition schematic shown in Figure 1.

Subsequently, the generated Chebyshev population $pop_{c}$, Tent population $pop_{T}$, Chebyshev refractive opposition population $pop_{c}^{o}$ and Tent refractive opposition population $pop_{T}^{o}$ are formed into a population of size 4·N, denoted as $pop_{total}$. Subsequently, the fitness function values of the $pop_{total}$ population individuals are calculated based on the objective function of the problem to be optimized; assuming that the solution problem is a minimization problem, the $pop_{total}$ population individuals are sorted in ascending order based on the fitness function values and the set of the top N individuals in the sorted order is used as the initialized population $pop=[X_{1},X_{2},\ldots ,X_{i},\ldots ,X_{N}]$ for subsequent iterations. By adjusting the initialization population for chaotic mapping and refraction opposition learning, the algorithm is equipped with higher solution space coverage in the initialization stage, which helps the algorithm to improve the global search performance in the later iteration stage and thus improves the algorithm’s multi-threshold image-segmentation accuracy.

### 3.2. Adaptive Learning Strategy

Zhang et al. [36] pointed out that individuals help the algorithm’s global search ability to improve by learning from the gaps of individuals possessing different properties, which in turn alleviates the local stagnation that the algorithm gets stuck in. Based on this inspiration, in order to alleviate the problem of insufficient optimization accuracy caused by the insufficient global exploration capability of HEOA in solving multi-threshold image-segmentation problems, this section proposes an adaptive learning strategy which aims to take into account the disparities associated with individuals of different natures while incorporating an adaptive factor in order to enhance the global exploration capability of the algorithm. The adaptive learning strategy will be described in detail in the following; firstly, the individual gaps considered in this study are the gap between the best individual and a better individual, the gap between the best individual and a worse individual, the gap between the better individual and the worse individual and the gap between two random individuals in the population; the four sets of gaps are expressed using Equation (19).

(19) $$\left\{\begin{array}{l} Gap_{1}=X_{best}-X_{better}\\ Gap_{2}=X_{best}-X_{worse}\\ Gap_{3}=X_{better}-X_{worse}\\ Gap_{4}=X_{rand1}-X_{rand2} \end{array}\right.$$

where $Gap_{1}$ denotes the gap between the best individual and the better individual, $Gap_{2}$ denotes the gap between the best individual and the worse individual, $Gap_{3}$ denotes the gap between the better individual and the worse individual and $Gap_{4}$ denotes the gap between the two random individuals. $X_{best}$ denotes the best individual in the population; $X_{better}$ denotes the better individual, defined as a random individual in the set of the first 5 individuals with the smallest value of the fitness function; $X_{worse}$ denotes the worse individual, defined as a random individual in the set of the first 5 individuals with the largest value of the fitness function; and $X_{rand1}$ and $X_{rand2}$ denote the two random individuals in the population that are not identical to each other. Subsequently, in order to reflect the degree of learnability associated with each set of gaps, the learnability of each set of gaps was determined using Equation (20).

(20) $$LF_{k}=\frac{\left\|Gap_{k}\right\|}{\sum_{k=1}^{4}\left\|Gap_{k}\right\|},(k=1,2,3,4)$$

where $LF_{k}$ denotes the degree of learnability of the kth set of gaps and “‖·‖” denotes the operation of modeling the vector. Subsequently, the learning ability of an individual needs to be taken into account; the smaller the value of the fitness function, the higher the quality of the representation of the individual, and in order to ensure its quality, the learning ability should be reduced, while, on the contrary, the larger the value of the fitness function, the greater the learning ability of the individual. Therefore, Equation (21) is used to represent the individual learning ability.

(21) $$SF_{i}=\frac{fit_{i}}{fit_{max}},\;(1\leq i\leq N)$$

where $SF_{i}$ denotes the learning ability of the ith individual and $fit_{max}$ denotes the value of the maximum fitness function in the population. Based on the above information, the learning process of the ith individual relative to the kth group of gaps is expressed using Equation (22).

(22) $$KA_{k}=e^{vl}\cdot \cos\;(2\cdot \pi \cdot (1-\frac{t}{Max_{iter}}))\cdot SF_{i}\cdot LF_{k}\cdot Gap_{k},(k=1,2,3,4)$$

where $KA_{k}$ denotes the amount of knowledge that the ith individual learns from the kth group of gaps, v is a constant used to change the shape of the spiral with a default value of 1 and l is a random number in the interval [−1, 1]. In addition to considering the degree of learnability of the gaps and the learning ability possessed by the individual, a cosine adaptive factor is also considered, which makes the learning process adaptive with the number of iterations of the algorithm. Subsequently, based on the above information, the state of the ith individual after learning from the four sets of gaps is expressed in Equation (23).

(23) $$X_{i}^{new}=X_{i}^{t}+KA_{1}+KA_{2}+KA_{3}+KA_{4}$$

Subsequently, the information for the ith individual is retained using Equation (24).

(24) $$X_{i}^{t+1}=\left\{\begin{array}{l} X_{i}^{new}if\;fit_{i}^{new}<fit_{i}\\ X_{i}^{t}otherwise \end{array}\right.$$

where $fit_{i}^{new}$ denotes the value of the fitness function of $X_{i}^{new}$. Individuals can ensure that the global exploration performance of the algorithm expands well and enhance the algorithm’s multi-threshold segmentation accuracy by learning from different gaps while combining the degrees of learnability associated with the gaps and their own learning ability.

### 3.3. Nonlinear Control Factor

**Figure 2:**
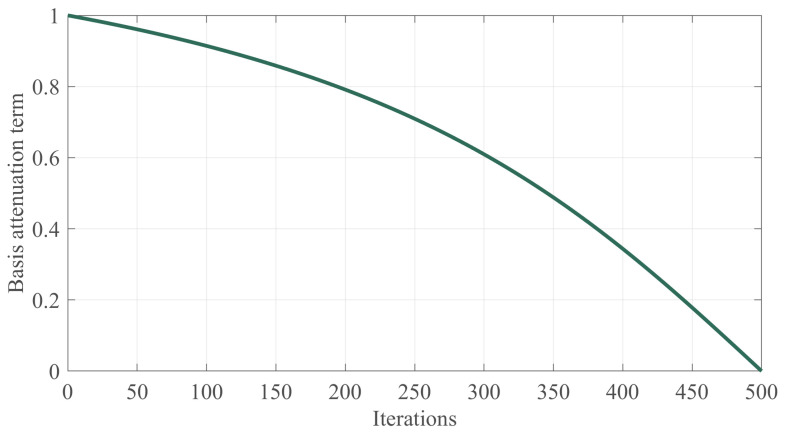
The basis attenuation term change chart.

**Figure 3:**
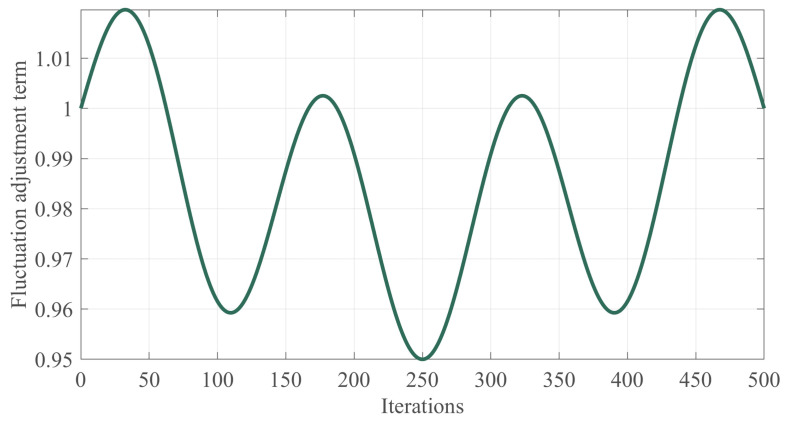
The fluctuation adjustment term change chart.

**Figure 4:**
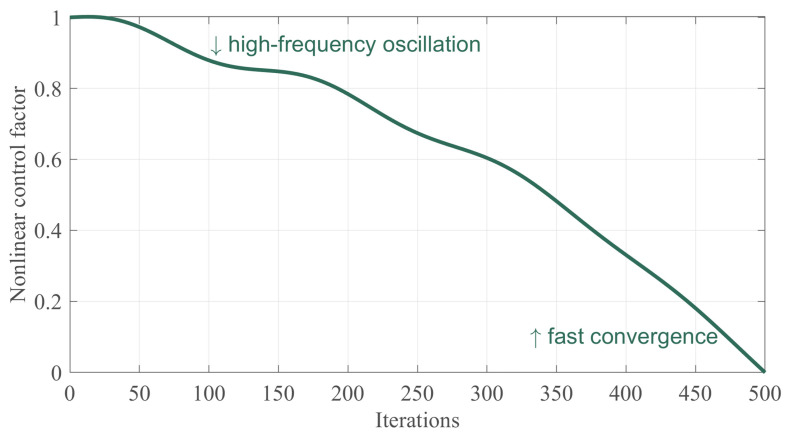
The nonlinear control factor change chart.

The original HEOA divides the global exploration phase and the development phase into fixed values, which is not conducive to the balance between global exploration and local exploitation in the iterative process of the algorithm, and results in the algorithm solving the multi-threshold image-segmentation problem being prone to falling into the local, suboptimal threshold problem. Mohamed et al. [37] pointed out that balancing these two phases by the nonlinear factor helps to enhance the balance between the two and improve the convergence accuracy of the algorithm. Therefore, based on this inspiration, a novel nonlinear control factor is proposed in this section to rationalize the global exploration phase and the local exploitation phase. Specifically, the proposed nonlinear control factor consists of a basis attenuation term incorporating an inverse tangent function and a fluctuation adjustment term incorporating sine and cosine functions, where the basis attenuation term is expressed as Equation (25).

(25) $$Base=\frac{arctan\left(s\cdot \left(1-\frac{t}{Max_{iter}}\right)\right)}{arctan(s)}$$

where arctan denotes the arc-tangent function and the parameter s denotes the decay rate factor, which in this study takes the value of a constant 3. Its trend with an increasing number of iterations is shown in Figure 2. In addition, the fluctuation adjustment term is calculated using Equation (26).

(26) $$Func=1+a\cdot \sin\left(b\cdot \pi \cdot \frac{t}{Max_{iter}}\right)\cdot \cos\left(c\cdot \pi \cdot \frac{t}{Max_{iter}}\right)$$

where a denotes the amplitude parameter, which takes the value of 0.05, and the frequency parameters b and c are used to adjust the fluctuation frequency, and take the values of 3 and 4, respectively, to generate asymmetric fluctuations; its trend with an increasing number of iterations is shown in Figure 3. Periodic fluctuations are introduced through the products of sine and cosine functions to ensure global exploration capability. Finally, the basis attenuation term and the fluctuation adjustment term are combined to form a nonlinear decay factor with attenuation as well as waviness to improve the balance between global exploration and local exploitation of the algorithm, as defined in Equation (27).

(27) $$\begin{array}{c} NCF=Base\cdot Func=\frac{arctan\left(s\cdot \left(1-\frac{t}{Max_{iter}}\right)\right)}{arctan(s)}\cdot \\ \left(1+a\cdot \sin\left(b\cdot \pi \cdot \frac{t}{Max_{iter}}\right)\cdot \cos\left(c\cdot \pi \cdot \frac{t}{Max_{iter}}\right)\right) \end{array}$$

where Figure 4 demonstrates the trend graph of the nonlinear control factor with the increase of the number of iterations, and from which it can be seen that the nonlinear factor decreases nonlinearly from 1 to 0. At the same time, notice, in the pre-iteration period, the specific certain fluctuating nature, which helps to ensure that the algorithm’s global search ability, and in the late iteration period, the specific fast decreasing tendency, which helps to ensure that the algorithm’s ability to exploit locally. Balancing the two stages by nonlinear control factors can significantly improve the convergence performance of the algorithm.

### 3.4. Three-Point Guidance Strategy Based on Bernstein Polynomials

When HEOA solves the multi-threshold image-segmentation problem, the algorithm suffers from the problem of insufficient local development ability and is unable to effectively exploit the solution region after locating the potentially optimal threshold region during the global search phase, which leads to decreases in the algorithm’s convergence speed and accuracy. Zhang et al. [38] pointed out that the guidance by the excellent individuals in the population can ensure that the local exploitation ability of the algorithm is effectively improved, improving the optimization accuracy of the algorithm. Based on this inspiration, a three-point guidance strategy based on Bernstein polynomials is proposed in this section, which combines Bernstein’s weighting property to weight three kinds of individuals with different attributes; the generated weighted individuals are used for individual guidance, which improves the algorithm’s ability relative to local exploitation while also ensuring a determinate global exploration capability. In the following, a three-point guidance strategy based on Bernstein polynomials will be described in detail. Firstly, the n-order Bernstein polynomials are defined by Equation (28).

(28) $$B_{w,n}(p)=C_{n}^{w}\cdot p^{w}\cdot {\left(1-p\right)}^{n-w}$$

where 0≤p≤1 denotes the probability of success of the sampling result, w denotes the number of instances associated with success in the sampling experiment, n denotes the total number of times the sampling experiment was carried out and $C_{n}^{w}$ denotes a binomial function, expressed as Equation (29).

(29) $$C_{n}^{w}=\frac{n!}{w!(n-w)!}$$

For the second-order Bernstein polynomials, we have n=2 and w=0,1,2, thus containing three polynomials, and by combining Equations (28) and (29), the second-order Bernstein polynomials are expressed within Equation (30).

(30) $$\left\{\begin{array}{l} B_{0,2}(p)={\left(1-p\right)}^{2}\\ B_{1,2}(p)=2\cdot p\cdot (1-p)\\ B_{2,2}(p)=p^{2} \end{array}\right.$$

**Figure 5:**
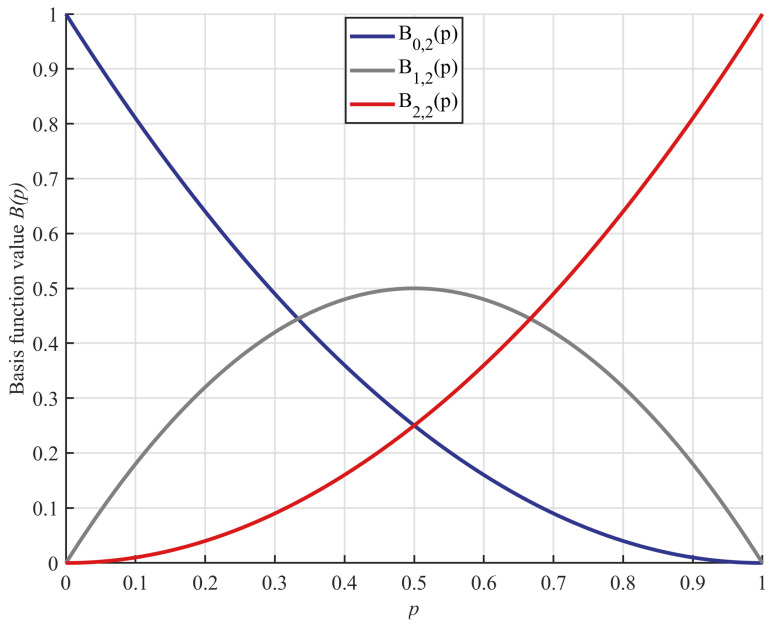
The graph of second-order Bernstein polynomials.

When p is incremented from 0 to 1, the functional image of the second-order Bernstein polynomial is shown in Figure 5, from which it can be found that the sum of $B_{0,2}(p)$, $B_{1,2}(p)$ and $B_{2,2}(p)$ is 1 when p is in the interval [0, 1]; this property will be utilized in the subsequent weighting operations for individuals with different attributes.

**Figure 6:**
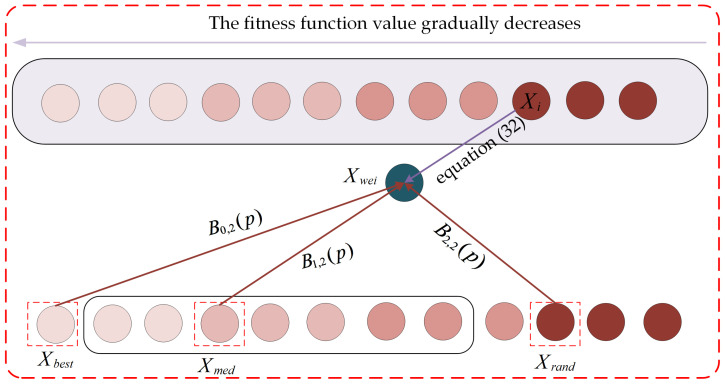
Simulation diagram for the three-point guidance strategy based on Bernstein polynomials.

In three-point guidance strategy based on Bernstein polynomials, the main consideration is the weighting of the best, median, and random individuals; this is performed using Equation (31).

(31) $$X_{wei}=B_{0,2}(p)\cdot X_{best}+B_{1,2}(p)\cdot X_{med}+B_{2,2}(p)\cdot X_{rand}$$

where $X_{wei}$ denotes the generated weighted individual, $X_{best}$ denotes the globally best individual, $X_{rand}$ denotes a random individual in the population and $X_{med}$ denotes the median individual, which is defined as a random individual in the set of individuals that have a smaller fitness function value than the current individual. Subsequently, after generating weighted individuals, the individual information is updated using Equation (32); the process is visualized in Figure 6.

(32) $$X_{i}^{new}=X_{i}^{t}+e^{\left(\frac{t}{Max_{iter}}\right)}\cdot randn\cdot (X_{wei}-X_{i}^{t})$$

where randn denotes a random number obeying a standard normal distribution. The individual information is then updated using Equation (24). By using the three-point guidance strategy based on Bernstein polynomials for individuals, the local exploitation of the algorithm is made possible, while also ensuring a certain population diversity and reducing the risk of falling into a local, suboptimal threshold.

### 3.5. Implementation of the CLNBHEOA

**Figure 7:**
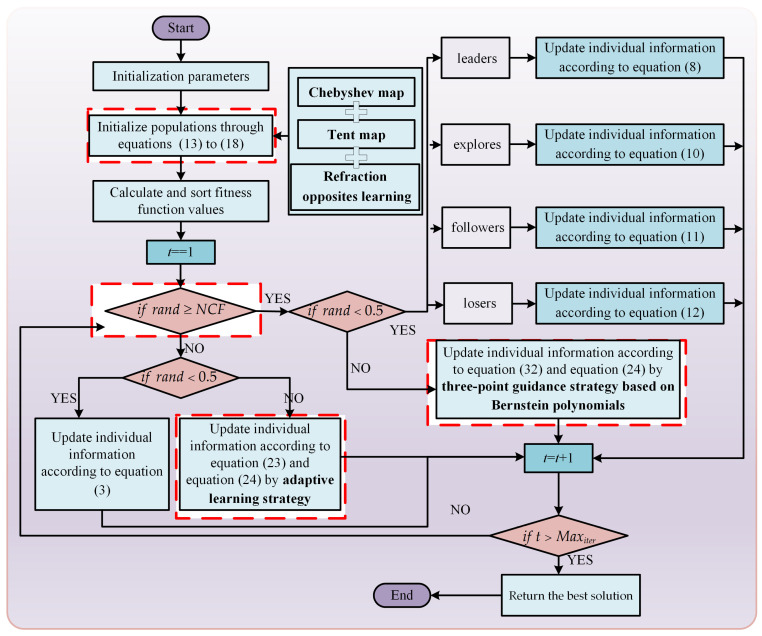
The CLNBHEOA flowchart.

In this section the main focus is on the execution logic of the proposed CLNBHEOA in solving the optimization problem. The CLNBHEOA is a novel algorithm proposed on the basis of HEOA algorithm, and which combines the improvement strategies. First, the Chebyshev–Tent chaotic mapping refraction opposites-based learning strategy is introduced in the initialization population stage to enhance the initial population quality of the algorithm and to improve the global search ability of the algorithm in the iterative process. Second, by introducing the adaptive learning strategy in the global exploration phase, the process effectively enhances the algorithm’s population diversity and reduces the risk of the algorithm falling into local, suboptimal thresholds. Furthermore, the two main phases of the algorithm are better coordinated by the introduction of a nonlinear control factor to balance the global exploration phase and the local exploitation phase of the algorithm. Finally, the introduction of a three-point guidance strategy based on Bernstein polynomials improves the effective exploitation performance of the algorithm and enhances the optimization accuracy of the algorithm. Compared with HEOA, it has better global search performance, as well as improved local exploitation performance, and its convergence accuracy is greatly improved. In order to better understand the execution process, Algorithm 2 gives the execution pseudo-code associated with the CLNBHEOA, and Figure 7 gives the flowchart of CLNBHEOA execution.
**Algorithm 2:** The pseudo-code associated with the CLNBHEOA
  **Input:** N, Dim, UB, LB, $Max_{iter}$, A=0.6
  **Output:** global best solution $X_{best}$
1. %Population initialization phase2. Generate initialized populations $pop=[X_{1},X_{2},\ldots ,X_{i},\ldots ,X_{N}]$ according to Equation (13) to Equation (18) by **the Chebyshev–Tent chaotic mapping refraction opposites-based learning strategy**, which is generated by integrating the Chebyshev map, the Tent map and the refraction opposites-based learning strategy.3. Calculate the fitness function value for the population $fit=[fit_{1},fit_{2},\ldots ,fit_{i},\ldots ,fit_{N}]$4. *for* $t=1:Max_{iter}$5. [~, ind] = sort(fit)6. $X_{best}=pop(ind(1))$7. *for* i=1:N8.    *if* rand<NCF9.       %Human exploration phase10.       *if* rand<0.511.         Update individual information according to Equation (3)12.       *else*13.         Update individual information according to Equations (23) and (24) by **adaptive**
      **learning strategy**14.       *end*15.    *else*16.       %Human development phase17.       *if* rand<0.518.         Categorized individuals into four roles based on the fitness function values fit: Leaders,         Explores, Followers, Losers19.         *if* individuals belong leaders20.          %Leaders update strategy21.          Update individual information according to Equation (8)22.         *end*23.         *if* individuals belong explores24.          %Leaders update strategy25.          Update individual information according to Equation (10)26.         *end*27.         *if* individuals belong followers28.          %Followers update strategy29.          Update individual information according to Equation (11)30.         *end*31.         *if* individuals belong losers32.          %Followers update strategy33.          Update individual information according to Equation (12)34.         *end*35.       *else*36.         Update individual information according to Equations (32) and (24) by **three-point**      **guidance strategy based on Bernstein polynomials**37.       *end*38.    *end*39. *end*40. *end*41. Output global best solution $X_{best}$

## 4. Experimental Procedure

In this section, the main focus is on explaining the procedure for the experiment. We will evaluate the optimization performance of the proposed CLNBHEOA by conducting experiments on the CEC2017 test function set equipped with 100 dimensions; the CEC2017 test-set specific information is shown in Table 1. It is also compared with nine novel and excellent algorithms to objectively and comprehensively evaluate the optimization performance of the CLNBHEOA; the parameter configuration information of these algorithms is shown in Table 2.

In order to ensure the fairness as well as repeatability of the experiments, the maximum number of function evaluations is set to 30,000, the population size is 30 and each group of experiments is independently and non-repeatedly executed 30 times to enhance the persuasive power of the experimental results. All of the experimental code was written and executed on MATLAB 2021Rb on a Windows 11 system, with the hardware conditions of 1 TB of solid-state hard disk and 32 Gb of operating memory.

## 5. Results

In this section, the optimization performance of the proposed CLNBHEOA on the CEC2017 test function and the multi threshold segmentation performance on six images are comprehensively evaluated.

### 5.1. Experimental Results on CEC2017 Test Function

In this section, the primary focus is on evaluating the optimization performance of the the CLNBHEOA on the CEC2017 test functions. Specifically, an analysis is conducted on various aspects of the algorithm’s optimization experiments, including population diversity, exploration/exploitation balance, fitness function value, stability, Wilcoxon rank-sum test, Friedman rank-sum test and convergence. These analyses confirm from multiple perspectives that the proposed CLNBHEOA in this study exhibits highly efficient optimization performance.

#### 5.1.1. Validation of Initialization Strategy Effectiveness

**Figure 8:**
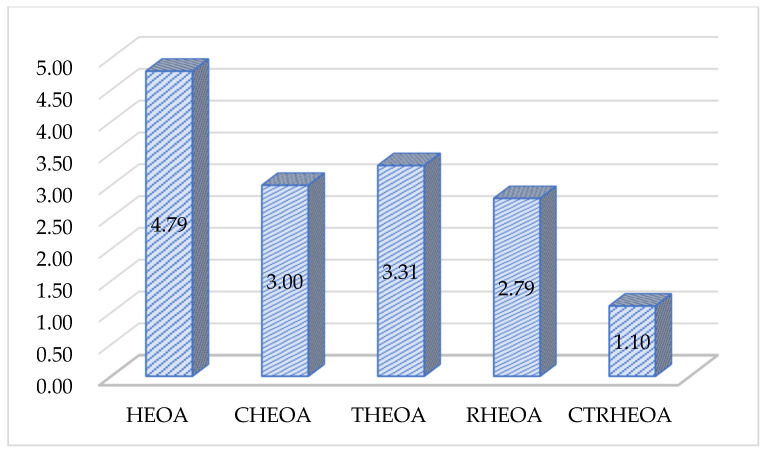
Friedman rank-sum test ranking.

The Chebyshev–Tent chaotic mapping refraction opposites-based learning strategy proposed in this paper mainly consists of two steps. Initially, a Chebyshev population of size N is initialized using the aforementioned Chebyshev chaotic strategy, while a Tent population of size N is initialized using the Tent chaotic strategy. Subsequently, refraction opposites-based learning is applied to the generated Chebyshev population to produce a refracted opposite Chebyshev population of size N; similarly, refraction opposites-based learning is applied to the generated Tent population to produce a refracted opposite Tent population of size N. The aforementioned steps collectively generate four populations, each of size N. Then, the top N individuals with the smallest fitness function values are selected from the combined set of 4·N individuals to form the initialization population in the iterative process of the algorithm. All the aforementioned steps constitute the Chebyshev–Tent chaotic mapping refraction opposites-based learning strategy proposed in this paper, which integrates the Chebyshev initialization strategy, the Tent initialization strategy and the refraction opposites-based learning strategy. Their combined application forms the Chebyshev–Tent chaotic mapping refraction opposites-based learning strategy proposed in this study. In this section, the enhancing effects of individual components within the Chebyshev–Tent chaotic mapping refraction opposites-based learning strategy on the algorithm’s performance are comprehensively validated. Specifically, the Chebyshev initialization strategy is introduced into the HEOA algorithm to form CHEOA, the Tent initialization strategy is introduced to form THEOA and the refraction opposites-based learning strategy is introduced to form RHEOA. The enhanced version of HEOA, formed by combining the aforementioned three strategies (i.e., the Chebyshev–Tent chaotic mapping refraction opposites-based learning strategy), is referred to as CTRHEOA. The four aforementioned schemes are employed to solve the CEC2017 test functions in order to evaluate the advantages of the Chebyshev–Tent chaotic mapping refraction opposites-based learning strategy proposed in this paper. The results of the Friedman rank-sum test are depicted in Figure 8. As can be observed from the figure, the average Friedman ranks of CHEOA, THEOA and RHEOA all surpass those of the HEOA algorithm, confirming the effectiveness of the three initialization strategies. Notably, CTRHEOA achieves superior solving performance compared to CHEOA, THEOA and RHEOA, indicating that the Chebyshev chaotic strategy, the Tent chaotic strategy and the refraction opposites-based learning strategy all contribute to the enhancement of the algorithm’s performance. However, when these three strategies are combined to form the Chebyshev–Tent chaotic mapping refraction opposites-based learning strategy, they can better promote the algorithm’s performance.

#### 5.1.2. Population Diversity Analysis

**Figure 9:**
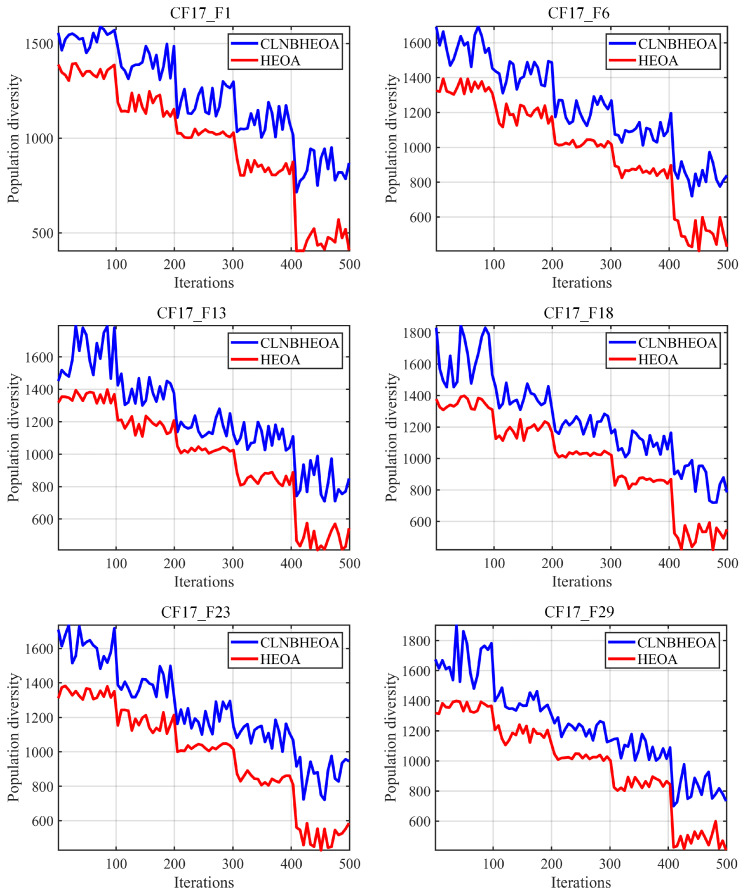
Algorithms for population diversity in CEC2017.

This section focuses on the analysis of the population diversity of the algorithm during the optimization process; higher population diversity means that the algorithm is more capable of jumping out of the local, suboptimal threshold, which helps the algorithm to improve the optimization accuracy. The experimental results are shown in Figure 9, where the X-axis represents the number of iterations, the Y-axis represents the population diversity of the algorithm during the running process, the red line represents the population diversity corresponding to HEOA and the blue line represents the population diversity corresponding to the CLNBHEOA.

As can be seen from the figure, on the simple modal test functions CF17_F1 and CF17_F6, compared with the original HEOA, the CLNBHEOA possesses higher population diversity throughout the iterative process, which enables the CLNBHEOA to possess a higher ability to jump out of the local, suboptimal threshold at the later stage of the iteration, which is mainly attributed to the proposed Chebyshev–Tent chaotic mapping refraction opposites-based learning strategy and adaptive learning strategy proposed in this paper, which can effectively enhance the population diversity of the algorithm and facilitate the global search process of the algorithm. In addition, on the complex multimodal functions CF17_F13, CF17_F18, CF17_F23 and CF17_F29, the CLNBHEOA still possesses higher population diversity throughout the iteration process compared to HEOA, which demonstrates that the strategy proposed in this paper is still able to enhance the algorithm’s ability to jump out of the local, suboptimal threshold effectively in the solving of complex multidimensional optimization problems. In summary, it can be concluded that the strategy proposed in this paper can significantly improve the population diversity during the execution of the CLNBHEOA, enhancing the global search performance and thus improving the optimization accuracy.

#### 5.1.3. Exploration/Exploitation Balance Analysis

**Figure 10:**
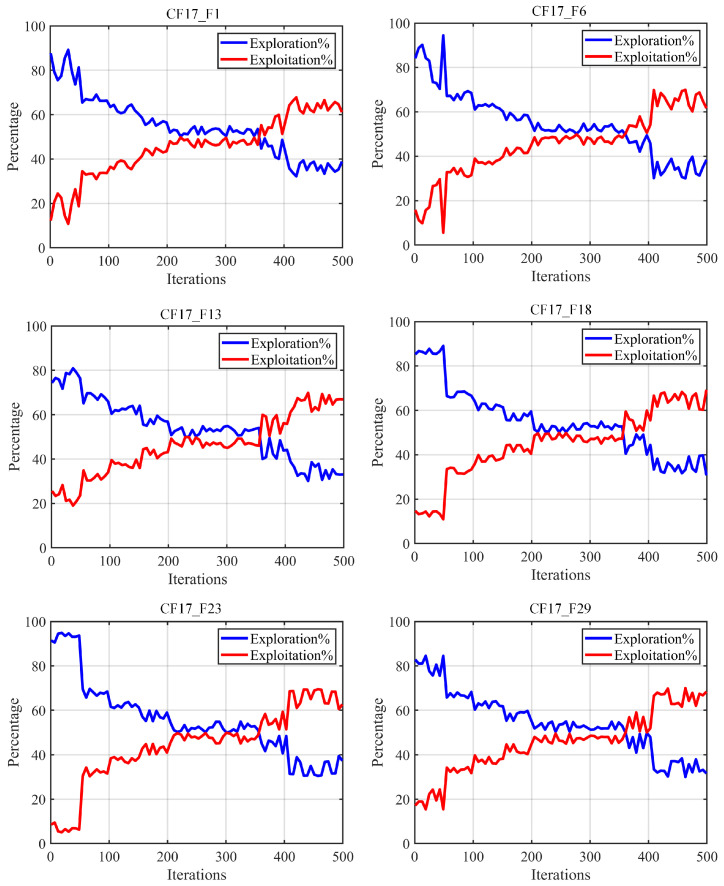
Algorithms’ balance of exploration/exploitation in CEC2017.

This section focuses on the analysis of the balance between the global exploration and local exploitation phases of the algorithm during the optimization execution. In the field of optimization, the balance between the global exploration and local exploitation phases is crucial; the typical optimization process used is to locate the potential optimal solution region by the global exploration phase first, and then use the local exploitation phase to further exploit the located potential optimal region, in order to improve the algorithm convergence accuracy and speed, so a reasonable balance between the two can help to improve the algorithm optimization accuracy. We used some representative CEC2017 test function sets to determine the exploration/exploitation ratios of the algorithms during execution, and the experimental results are shown in Figure 10, where the X-axis denotes the number of iterations, the Y-axis denotes the ratios, the blue line denotes the global exploration rate and the red line denotes the local exploitation rate.

As can be seen from the figure, based on the statistics associated with the CEC2017 test function set, the algorithm possesses a high global exploration rate at the early stage of iteration, which is mainly due to the fact that the Chebyshev–Tent chaotic mapping refraction opposites-based learning strategy and adaptive learning strategy effectively improves the algorithm’s global search capability, which makes it tend to perform global exploration behavior in the early stage; it can then effectively locate more potential optimal regions. In the middle of iteration, the global exploration rate and local exploitation rate tend to balance, which is mainly due to the introduction of the nonlinear control factor described in this paper, which makes the global exploration and local exploitation behaviors balanced and can effectively improve the optimization accuracy of the algorithm to avoid the phenomenon of local stagnation. In the later stage of iteration, the algorithm tends to perform local exploitation operations which depend on the three-point guidance strategy based on Bernstein polynomials proposed in this paper; these enhance the algorithm’s local exploitation capability, resulting in great improvements in convergence speed and convergence accuracy, while at the same time, the algorithm maintains a 35% or so global exploration rate, which helps to ensure the algorithm’s ability to jump out of the local, suboptimal threshold. In summary, the CLNBHEOA proposed in this paper achieves a good balance between its global exploration phase and local exploitation phase due to the advanced nature of the strategy, which helps to improve the optimization accuracy of the algorithm.

#### 5.1.4. Fitness Function Value Analysis

In this section, the optimization performance of the CLNBHEOA and nine comparison algorithms on the 100-dimensional CEC2017 test function set is comprehensively evaluated, and the optimization performance of the CLNBHEOA is objectively evaluated through the statistical analysis of the mean and standard deviation of the numerical results of 30 independent experiments; the experimental results are shown in Table 3, where ”Mean Rank” denotes the average numerical rank of the algorithm on the test function set and “Final Rank” denotes the final rank based on the Mean Rank information.

**Figure 11:**
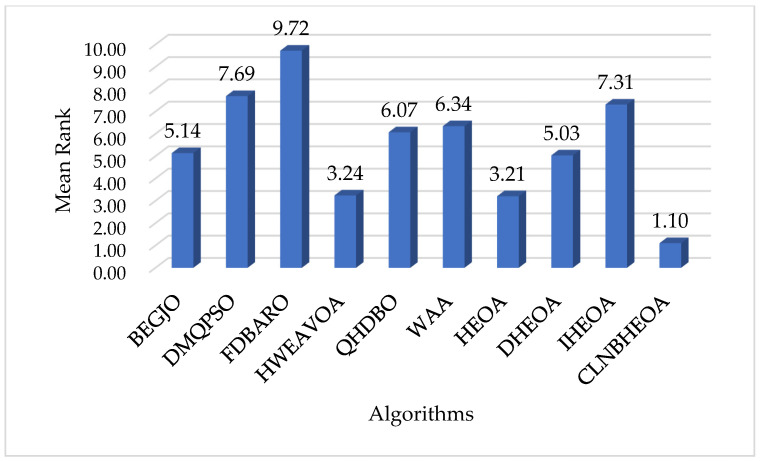
Algorithms’ mean ranking for CEC2017.

**Figure 12:**
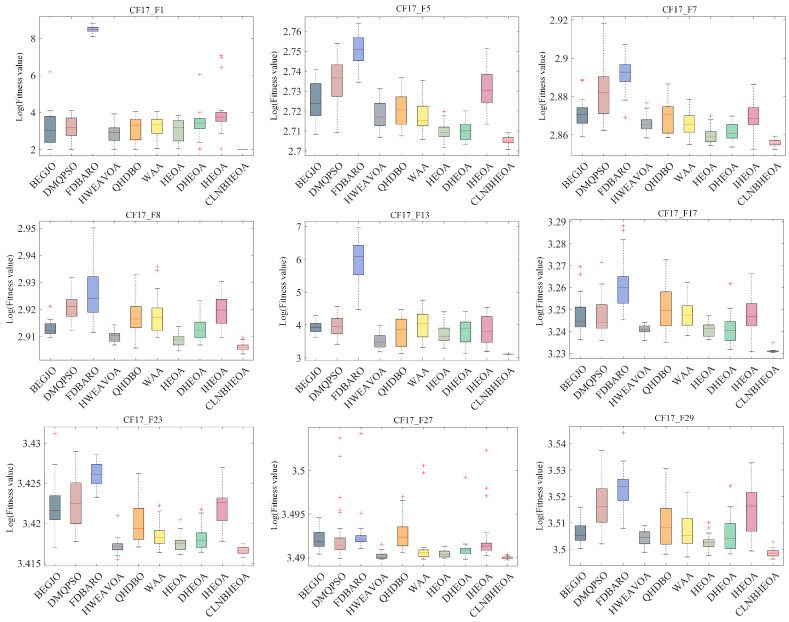
Algorithms’ box plots for CEC2017.

As can be seen from the table, when solving the simple multimodal optimization problems CF17_F1 to CF17_F10, compared with the comparison algorithms, the CLNBHEOA ranked first on nine test functions, with a winning rate of 100%, which is mainly due to the fact that the three-point guidance strategy proposed in this paper based on Bernstein polynomials effectively improves the local exploitation ability of the algorithm and effectively improves the optimization accuracy of the algorithm. In addition, when solving the complex multimodal optimization problems CF17_F11 to CF17_F30, compared with the comparison algorithms, the CLNBHEOA ranked first on 17 test functions with a winning rate of 85%, although it ranked second on CF17_F20, CF17_F21 and CF17_28, which is a weaker result than seen with the comparison algorithms, but it possesses a stronger ability to solve the multimodal optimization problems in a comprehensive perspective, given its modal optimization problem-solving ability. This is mainly due to the Chebyshev–Tent chaotic mapping refraction opposites-based learning strategy and adaptive learning strategy proposed in this paper to enhance the global search performance of the algorithms, and enhance the ability to jump out of a locally optimal solution. It also combines the nonlinear control factor to provide a good balance between the global exploration phase and the local exploitation phase, enabling it to solve multimodal optimization problems effectively. As can be seen from the last row of the table, the CLNBHEOA outperforms the original HEOA and the comparison algorithms, with a mean ranking of 1.10 on the 29 tested functions. To better represent the gap in algorithmic performance, Figure 11 demonstrates the average ranking histograms, with the CLNBHEOA being associated with lower column heights. In addition to the optimization accuracy, the solution stability is also very important; higher stability can ensure that the algorithm has better applicability to the real environment. Figure 12 shows some box plots of the algorithm associated with solving the CEC2017 test function, from which it can be seen that, in most cases, the CLNBHEOA has a higher degree of solution aggregation, and also has fewer anomalies, which indicates that the proposed algorithm has a higher degree of solution stability.

In summary, it can be concluded that the CLNBHEOA proposed in this paper, due to the improvement of HEOA from the perspectives of global exploration, local exploitation and two-phase equilibrium, improves the optimization accuracy in solving simple modal and complex multimodal optimization problems, and in addition, it has higher solution stability and practical applicability.

#### 5.1.5. Expansion Analysis of Fitness Function Values

In this section, an extensive analysis is primarily performed on the fitness function values of the proposed CLNBHEOA relative to its handling of varying test dimensions and different maximum numbers of function evaluations. To achieve this, the test dimension is set to 30, and the maximum number of function evaluations is set to 300,000. Each experiment is independently conducted 30 times without repetition, in order to compile the experimental results. The evaluation metrics encompass the mean and standard deviation, with the best values emphasized in bold. The experimental results on the CEC2017 test functions are presented in Table 4.

As can be seen from the table, the CLNBHEOA achieved first place for both unimodal functions F1 and F3, confirming that the introduction of the three-point guidance strategy based on Bernstein polynomials in this paper enhances the algorithm’s exploitative capability, thereby improving local optimization precision. Additionally, on simple multimodal functions F4 through F10, the CLNBHEOA attained a win rate of over 90%, with MS-TSA ranking first on function F6. Overall, due to the CLNBHEOA’s superior global exploration ability, it demonstrates relatively higher performance in solving simple multimodal problems. On complex multimodal functions F11 through F30, the CLNBHEOA achieved optimal optimization accuracy on 17 test functions, with a win rate of 85%, indicating that the CLNBHEOA possesses a higher level of balance between global exploration and local exploitation, enabling it to effectively develop local regions and enhance algorithm precision. Furthermore, from a comprehensive perspective, in the tests of 29 test functions, the CLNBHEOA achieved an average ranking of 1.1, ranking first overall and surpassing the second-ranked HWEAVOA algorithm by 61.1%. In summary, the CLNBHEOA proposed in this paper can be considered an effective method for solving high-dimensional complex optimization problems.

#### 5.1.6. Nonparametric Rank-Sum Test Analysis

**Figure 13:**
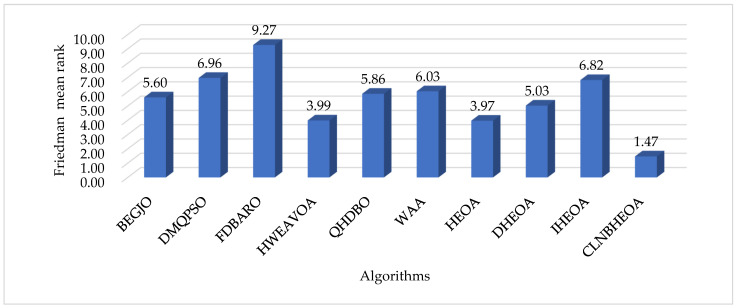
The algorithm’s Friedman rank values (CEC2017).

The previous subsection analyzed the numerical results of the algorithm in solving the CEC2017 test function set, but the results of the numerical analysis may be affected by outliers. In order to assess the optimization performance of the CLNBHEOA more comprehensively and to reduce the chance of the results, nonparametric rank-sum tests, including the Wilcoxon rank-sum test with a significance factor of 0.05 and the Friedman rank-sum test, are performed on the results of 30 independent experiments in this section. In the Wilcoxon rank-sum test, the use of the symbol “+” indicates that the comparison algorithm performance is significantly better than the CLNBHEOA, “=” indicates that the comparison algorithm performance is not significantly different from the CLNBHEOA and “−” indicates that the comparison algorithm performance is significantly weaker than that of the CLNBHEOA. The results of the Wilcoxon rank-sum test experiment are shown in Table 5 and the results of the Friedman rank-sum test experiment are shown in Figure 13.

As can be seen from the table, the CLNBHEOA, in the Wilcoxon rank-sum test, demonstrated a significance factor of 0.05, significantly outperforming DMQPSO, FDBARO, QHDBO, WAA and DHEOA on 29 test functions, and achieving significantly better results than HWEAVOA and HEOA on 28 test functions and significantly outperforming BEGJO on 27 test functions, with a win rate of over 93.1%, ending ranked first overall. This is mainly due to the fact that the learning strategies proposed in this paper improve the performance of the algorithm from the perspective of global exploration capability as well as local exploitation capability. In addition, as can be seen in Figure 13, the CLNBHEOA achieved a mean ranking of 1.47 on the Friedman rank-sum test, with a significance factor of 0.05. Compared to the second-ranked HEOA, its ranking was improved by 62.9%, which confirms that the CLNBHEOA proposed in this paper possesses higher multimodal optimization problem-solving performance and can be considered as an effective optimization-related problem-solving tool. In summary, this section confirms that the CLNBHEOA possesses efficient performance, given the nonparametric tests performed and the experimental results, and it also confirms that the four proposed improvement schemes demonstrate positive contributions in the context of the algorithm performance improvement.

#### 5.1.7. Convergence Analysis

**Figure 14:**
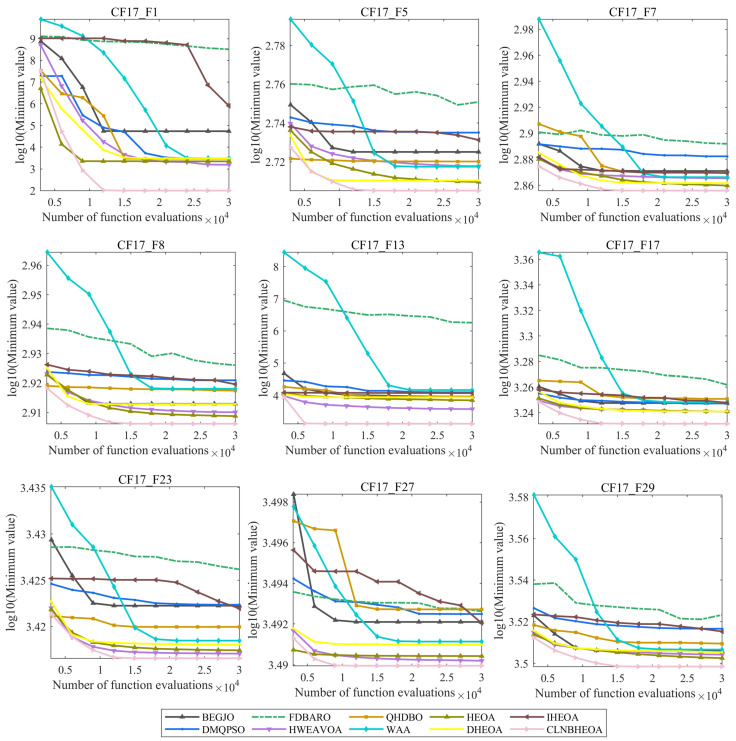
Convergence curves of the algorithm (CEC2017).

The above subsection confirms that the CLNBHEOA possesses efficient optimization performance from the point of view of numerical results as well as non-parametric tests, but in addition to this, the convergence nature of the algorithm is also very important, as it directly affects the applicability of the algorithm in real-world scenarios. Figure 14 illustrates the convergence plot of the algorithm solution process, where the x-axis represents the number of function evaluations and the vertical coordinate represents the logarithmic value of the fitness function value.

As can be seen from the figure, when solving the simple multimodal optimization problem, the algorithm has a certain leading edge at the early stage of iteration due to the introduction of the Chebyshev–Tent chaotic mapping refraction opposites-based learning strategy by the CLNBHEOA in the initialization phase of the population. After the 9000th function evaluation, the CLNBHEOA tends to converge, and the convergence accuracy is ahead of the comparative algorithms, and demonstrates faster convergence speed. This is mainly due to the introduction of the adaptive learning strategy, nonlinear control factor and three-point guidance strategy based on Bernstein polynomials, which improves the algorithm’s abilities of global exploration and local exploitation to a great extent and also strengthens its ability of jumping out of the local region, which improves the convergence speed of the algorithm. Moreover, when solving complex multimodal optimization problems, the advantages of the CLNBHEOA’s initialization population scheme provide a good convergence advantage at the beginning of the iteration. As the algorithm iterates, it converges after about 15,000 function evaluations, and its convergence accuracy is ahead of those of the comparison algorithms, while also having a higher convergence speed. Since the CLNBHEOA has stronger global exploration capability, the algorithm is more capable of solving multimodal problems. In summary, we can conclude that the CLNBHEOA proposed in this paper possesses higher convergence speed and accuracy in solving complex multimodal optimization problems and can be considered an optimization tool of high practicality.

### 5.2. Experimental Results on Multi-Threshold Image Segmentation

The multi-threshold image-segmentation problem is mainly concerned with finding the best segmentation threshold in order to segment the image into multiple regions, so that the researchers can better analyze the different attribute regions of the image, and ultimately better extract the image information, as well as perform better quantitative analysis of the image. In this section, the best segmentation thresholds are mainly identified using the Otsu method, the main idea of which is to determine the best segmentation thresholds by taking the pixels of the original image as inputs to the algorithm, and subsequently maximize the levels of differences between the different regions. Meanwhile, in order to effectively overcome the problem of traditional search methods, which are prone to fall into local best thresholds when the number of thresholds increases, multi-threshold segmentation is modeled as an optimization problem, and the interclass variance of Otsu’s method is used as the objective function; then, the CLNBHEOA proposed in this paper is used to efficiently search for the best combination of thresholds to improve the quality and efficiency of multi-threshold image segmentation. The concept of the Otsu method and the evaluation criteria used for solving multi-threshold image segmentation using the CLNBHEOA will be described in detail in the subsequent subsections.

#### 5.2.1. The Concept of Otsu Method

In this section, the concept of the Otsu method is introduced. Otsu’s approach is to identify the best segmentation threshold based on the differences between different segmentation regions. Suppose there is an image I with a total of L gray levels and the number of pixels in gray level i is $n_{i}$; it can be deduced that the total number of pixels in image I is N, which is expressed in Equation (33).

(33) $$N=\sum_{i=0}^{L-1}n_{i}$$

Then, the distribution probability of gray level i can be derived as $P_{i}$, as expressed in Equation (34).

(34) $$P_{i}=\frac{n_{i}}{N},\;\;\;\;i=0,1,\ldots ,L-1$$

where $p_{i}\geq 0,\;\;p_{0}+p_{1}+\cdots +p_{l-1}=1$. Assuming that the number of segmentation thresholds is set to k, if the gray value t is confirmed as the segmentation threshold, the threshold t divides the image into two regions; the pixel region with gray level [1,t] is the target region, and the pixel region with gray level [t+1,L−1] is the background region. Assuming that the ratio of the number of pixels in the target region to the total pixels is $\omega_{0}$ and the average gray level is $\mu_{0}$, the ratio of the number of pixels in the background region to the total pixels is $\omega_{1}$, the average gray level is $\mu_{1}$, the total average gray value of the image is μ and the variance between the different regions is ν. $\omega_{0}$, $\mu_{0}$, $\omega_{1}$, $\mu_{1}$, μ and ν are obtained by Equations (35)–(40).

(35) $$\omega_{0}=\sum_{i=0}^{t}P_{i}$$

(36) $$\mu_{0}=\sum_{i=0}^{t}\frac{iP_{i}}{\omega_{0}}$$

(37) $$\omega_{1}=\sum_{i=t+1}^{L-1}P_{i}$$

(38) $$\mu_{1}=\sum_{i=t+1}^{L-1}\frac{iP_{i}}{\omega_{1}}$$

(39) $$\mu =\sum_{i=0}^{k-1}\omega_{i}\mu_{i}=\sum_{i=0}^{L-1}iP_{i}$$

(40) $$v(t)=\omega_{0}{\left(\mu_{0}-\mu \right)}^{2}+\omega_{1}{\left(\mu_{1}-\mu \right)}^{2}=\omega_{0}\omega_{1}{\left(\mu_{0}-\mu_{1}\right)}^{2}$$

The best segmentation threshold, $t_{best}$, is calculated using Equation (41).

(41) $$t_{best}=\arg\max_{0\leq t\leq L-1}[v(t)]$$

Similarly, the k threshold class variance is calculated using Equation (42).

(42) $$\begin{array}{cc} v(t_{1},t_{2},\ldots ,t_{k}) & =\omega_{0}\omega_{1}{\left(\mu_{0}-\mu_{1}\right)}^{2}+\omega_{0}\omega_{2}{\left(\mu_{0}-\mu_{2}\right)}^{2}\\ & +\cdots +\omega_{0}\omega_{k}{\left(\mu_{0}-\mu_{k}\right)}^{2}+\omega_{1}\omega_{2}{\left(\mu_{1}-\mu_{2}\right)}^{2}\\ & +\cdots +\omega_{1}\omega_{3}{\left(\mu_{1}-\mu_{3}\right)}^{2}+\cdots +\omega_{k-1}\omega_{k}{\left(\mu_{k-1}-\mu_{k}\right)}^{2} \end{array}$$

where $\omega_{i}$ and $\mu_{i}$ are calculated as Equations (43) and (44), respectively.

(43) $$\omega_{i-1}=\sum_{i=t_{i-1}+1}^{t_{i}}P_{i},\;\;1\leq i\leq k+1$$

(44) $$\mu_{i-1}=\sum_{i=t_{i-1}+1}^{t_{i}}\frac{iP_{i}}{\omega_{i-1}},\;\;1\leq i\leq k+1$$

Assuming that the best threshold of Equation (42) is $T_{best}=(t_{1}^{*},t_{2}^{*},\ldots ,t_{k}^{*})$, the best threshold, $T_{best}$, is calculated by Equation (45)

(45) $$T_{best}=\arg\max_{0\leq t_{1}\leq t_{2}\leq \cdots \leq t_{k}}\left[\nu \left(t_{1},t_{2},\ldots ,t_{k}\right)\right]$$

#### 5.2.2. Experimental Results Analysis

**Figure 15:**
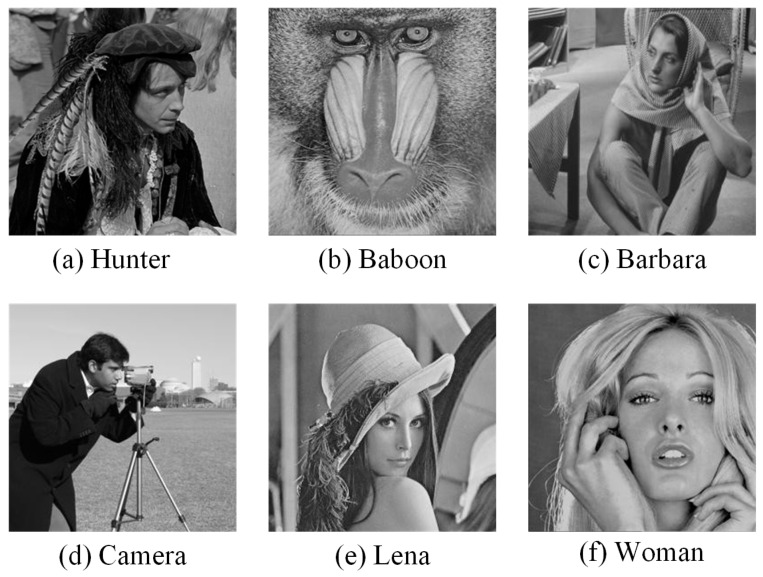
Six benchmark images.

In this section, the performance of the CLNBHEOA in solving image-segmentation problems is analyzed. Specifically, the CLNBHEOA is compared experimentally with five comparison algorithms; the algorithm parameter information is set as shown in Table 6, the maximum number of iterations is set to 100, the population size is set to 40, each experiment is independently and non-repeatedly executed 20 times, and the experiments are conducted on six images; the image-related information is shown in Figure 15. Peak Signal to Noise Ratio (PSNR) [49], Structural Similarity (SSIM) [50] and Feature Similarity (FSIM) [51] are analyzed to comprehensively evaluate the performance of the algorithm in solving the image-segmentation problem. Among them, the higher the value of PSNR and SSIM, the lower the distortion in the segmented image and the higher the quality of segmented image and the higher the value of FSIM, the lower the classification error rate.

**Figure 16:**
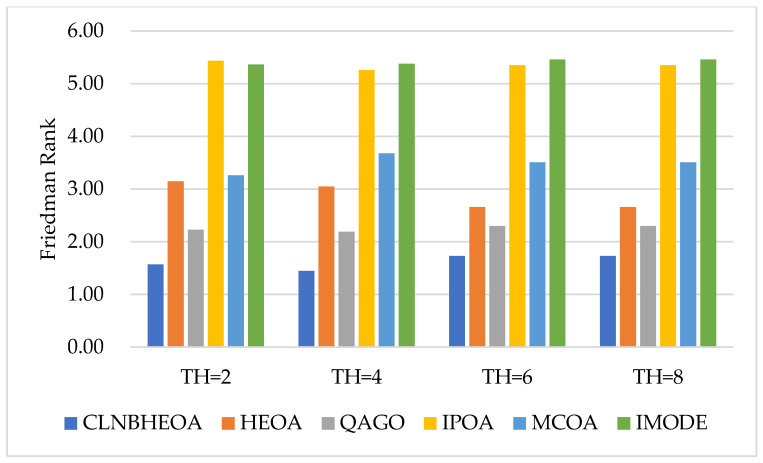
Friedman rank values for fitness function values of algorithms on multi-threshold image-segmentation problems.

In this case, the number of segmentation thresholds was set experimentally to the four values of 2, 4, 6 and 8, and the results of the algorithm for the experiment on six images for the fitness function are shown in Table 7, where “Friedman Rank” denotes the Friedman mean test ranking of the fitness function values for 20 independent experiments and the ”Rank” denotes the final ranking based on “Friedman Rank”; Figure 16 visualizes the Friedman mean test ranking on the fitness function values. As can be seen from the table, compared to the comparison algorithms, the CLNBHEOA obtained the top rank for the average fitness function values in the 24 experiments conducted using different numbers of segmentation thresholds, with a percentage of 100%, and achieving excellent performance. This is mainly due to the fact that the four learning strategies proposed in this paper update the performance of the algorithm from different perspectives, allowing it to have a higher optimal threshold finding capability. Meanwhile, in the Friedman mean test performed, the CLNBHEOA achieved an average ranking of 1.62, which is 28% ahead of the second-ranked QAGO, with an average ranking of 2.25. Meanwhile, as can be seen in Figure 16, the CLNBHEOA scored first place in the experiments with different numbers of segmentation thresholds. In summary, it can be concluded that the CLNBHEOA is an efficient image-segmentation method.

**Figure 17:**
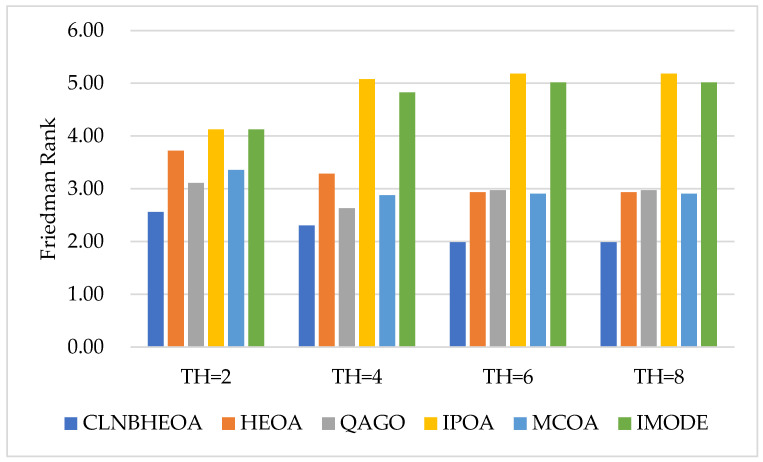
Friedman ranks for PSNR values of the algorithms for multi-threshold image-segmentation problems.

In addition, the experimental results of PSNR metrics on the six images are shown in Table 8, where “Friedman Rank” denotes the Friedman mean test ranking of the fitness function values for 20 independent experiments, and “Rank” denotes the final ranking based on “Friedman Rank”; Figure 17 visualizes the Friedman mean test rankings as to the PSNR values. From the table, it can be seen that the CLNBHEOA scored first place in 23 out of 24 image-segmentation experiments with the number of thresholds set at 2, 4, 6 and 8, with a winning rate of 95.8%. This means that the lower the degree of frame-loss in the segmented image, the better the image-segmentation performance. In addition, it can also be seen from the last row of the table that the CLNBHEOA achieved a Friedman’s mean test ranking of 2.21, which is 24.3% ahead of the second-ranked QAGO’s 2.92. Meanwhile, as can be seen in Figure 17, the CLNBHEOA ranked first in PSNR metrics in all the different threshold number segmentation experiments, demonstrating its efficient image-segmentation capability.

**Figure 18:**
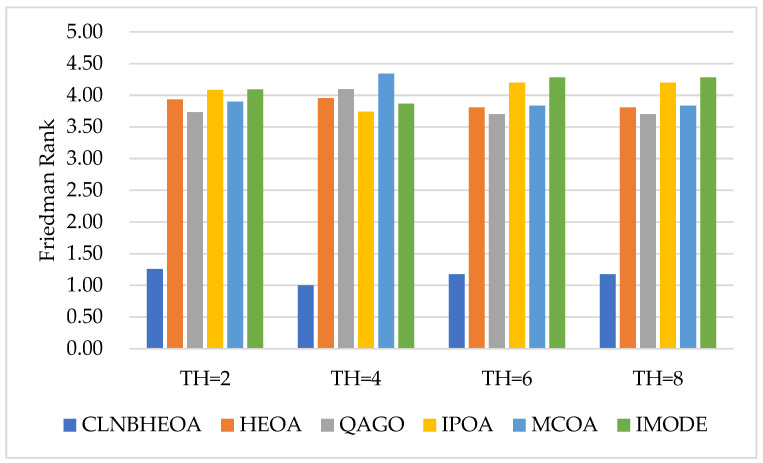
Friedman rank for the SSIM values of algorithms relative to multi-threshold image-segmentation problems.

Meanwhile, the experimental results of SSIM metrics on the six images are shown in Table 9, where “Friedman Rank” denotes the Friedman mean test ranking of the fitness function values for 20 independent experiments and “Rank” denotes the final ranking based on “Friedman Rank”; Figure 18 visualizes the Friedman mean test rankings for the SSIM values. From the table, it can be seen that the CLNBHEOA scores first place in 24 experiments of image segmentation with the number of thresholds set at 2, 4, 6 and 8, which is a 100% winning rate, compared to the comparison algorithms. This means that the segmented image possesses higher original similarity and that the algorithm shows strong image-segmentation performance. In addition, from the last row of the table, it can be seen that the CLNBHEOA achieved a Friedman mean test ranking of 1.15, which is 69.8% ahead of the second-ranked QAGO, with 3.81. Meanwhile, as can be seen in Figure 18, the CLNBHEOA ranks first in SSIM metrics in all the different threshold number segmentation experiments, indicating that it is associated with higher structural similarity.

**Figure 19:**
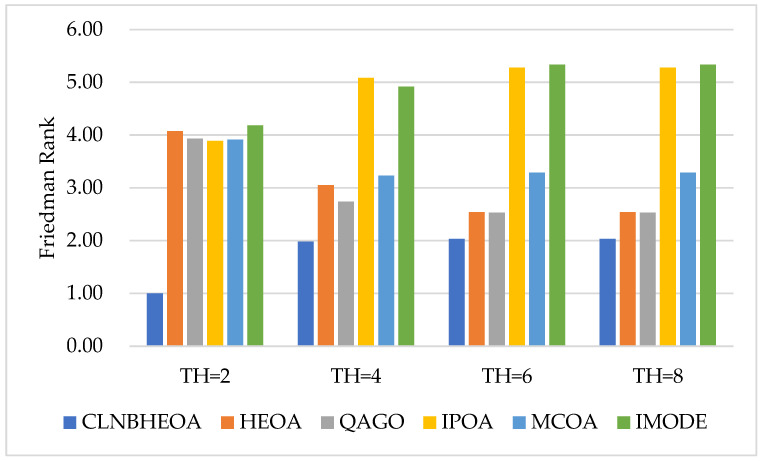
Friedman rank for the FSIM values of algorithms in the multi-threshold image-segmentation problems.

Meanwhile, the experimental results for the FSIM metrics on the six images are shown in Table 10, where “Friedman Rank” denotes the Friedman mean test ranking of the fitness function values for 20 independent experiments and “Rank” denotes the final ranking based on “Friedman Rank”; Figure 19 visualizes the Friedman mean test rankings for the FSIM values. From the table, it can be seen that in 24 image-segmentation experiments with the number of thresholds set at 2, 4, 6 and 8, the CLNBHEOA obtains first place in 24 experiments, with a winning rate of 100%, compared to the comparison algorithms. This implies that the segmented image possesses higher feature similarity, and that the algorithm has a lower classification error rate and demonstrates strong image-segmentation performance. In addition, from the last row of the table, it can be seen that the CLNBHEOA achieved a Friedman mean test ranking of 1.76, which is 39.9% ahead of the second-ranked QAGO, with 2.93. Meanwhile, as can be seen in Figure 19, the CLNBHEOA ranked first in the FSIM metrics in all the different threshold number segmentation experiments, which is an indication of possessing higher feature similarity and a lower classification error rate.

In summary, it is confirmed that the CLNBHEOA has a lower degree of image distortion and a lower classification error rate in solving the multi-threshold image-segmentation problem; this is found by analyzing the values of the fitness function, the peak signal-to-noise ratio (PSNR), the structural similarity (SSIM) and the feature similarity (FSIM). In that this enables the segmentation of the image while maximizing the retention of the image quality, the CLNBHEOA can be considered an effective multi-threshold image-segmentation method.

## 6. Discussion

In this paper, to address the shortcomings of the HEOA algorithm, such as insufficient global exploration capability, inadequate local exploitation capability and the resulting imbalance that leads to susceptibility to local optima-based traps and diminished optimization precision, the Chebyshev–Tent chaotic mapping refraction opposites-based learning strategy, the adaptive learning strategy, the nonlinear control factor and the three-point guidance strategy based on Bernstein polynomials were specifically introduced to enhance the performance of the algorithm, thereby forming the CLNBHEOA. The proposed CLNBHEOA underwent performance testing as to CEC2017 functions. The experimental results indicate that under varying dimensionalities and different function evaluation counts, compared to the most recent advanced variants and the latest improvements of the HEOA algorithm, the CLNBHEOA achieved a win rate exceeding 90% across different modal functions, demonstrating its promising potential as an optimization method. Subsequently, the CLNBHEOA was employed to solve practical image-segmentation problems. Through comparison with six high-performance algorithms, including the champion algorithm IMODE, it was confirmed that the CLNBHEOA is an effective tool for image segmentation, achieving a win rate exceeding 95% in terms of fitness function values, PSNR, SSIM and FSIM metrics and exhibiting superior performance compared to the competing algorithms.

## 7. Conclusions

In this study, an enhanced HEOA algorithm, called CLNBHEOA, is proposed, utilizing the combination of four learning strategies to address the poor segmentation effect of HEOA when dealing with multi-threshold image segmentation. Firstly, using the Chebyshev–Tent chaotic mapping refractive contrastive learning strategy gives the initial population a better ability to traverse the solution space. Secondly, an adaptive learning strategy is proposed, which improves the ability of the algorithm to jump out of the locally optimal threshold by learning from individual information gaps possessing different properties, while incorporating adaptive factors. In addition, the nonlinear control factor is proposed to better balance the global exploration phase and the local development phase of the algorithm. Finally, the three-point bootstrap strategy based on Bernstein polynomials is proposed to enhance the local exploitation of the algorithm. Subsequently, it is confirmed that the CLNBHEOA has higher global search capability and search performance due to the introduction of the strategy in this paper; this is found by performing optimization performance tests on the CEC2017 test function set, in which it surpasses the comparative algorithms by more than 90% in terms of the fitness function value metric. At the same time, the CLNBHEOA, with excellent performance, is applied to solve a multi-threshold segmentation problem using six images; the experiments show that it has higher structural similarity and feature similarity in solving the image-segmentation problem; it achieves a win rate of over 95% in terms of the fitness function value, PSNR, SSIM and FSIM metrics, and has higher image-segmentation performance. However, despite the proposed CLNBHEOA achieving superior performance in most scenarios, it still exhibits certain limitations when tackling specific optimization problems. Additionally, the performance of the CLNBHEOA has only been assessed thus far on the CEC2017 test function suite and multi-threshold image-segmentation problems, which merely validates its effective applicability to similar multimodal continuous optimization problems and combinatorial optimization issues. Its applicability in discrete optimization problems and high-dimensional combinatorial optimization remains unverified. Consequently, to further explore its applicability in other domains, our subsequent work plan is outlined as follows.

In the subsequent work, we will focus on the following three points: 1. Targeted learning strategies are proposed for specific types of optimization problems to strengthen the advantages of the algorithms. 2. Work is proposed to investigate the applicability of the CLNBHEOA in high-dimensional combinatorial optimization problems similar to parameter identification issues in photovoltaic systems [56,57], thereby addressing the existing limitations regarding its application domains. 3. The CLNBHEOA is to be extended to multi-objective models to further address the challenges of multi-objective combinatorial optimization problems.

## Figures and Tables

**Table 1:** The CEC2017 functions test set.

Functions	Types	Name	Best
CF17_F1	Unimodal	Shifted and Rotated Bent Cigar Function	100
CF17_F3		Shifted and Rotated Zakharov Function	300
CF17_F4	Multimodal	Shifted and Rotated Rosenbrock’s Function	400
CF17_F5		Shifted and Rotated Rastrigin’s Function	500
CF17_F6		Shifted and Rotated Expanded Scaffer’s F6 Function	600
CF17_F7		Shifted and Rotated Lunacek Bi-Rastrigin Function	700
CF17_F8		Shifted and Rotated Non-Continuous Rastrigin’s Function	800
CF17_F9		Shifted and Rotated Levy Function	900
CF17_F10		Shifted and Rotated Schwefel’s Function	1000
CF17_F11	Hybrid	Hybrid function 1 (N = 3)	1100
CF17_F12		Hybrid function 1 (N = 3)	1200
CF17_F13		Hybrid function 3 (N = 3)	1300
CF17_F14		Hybrid function 4 (N = 4)	1400
CF17_F15		Hybrid function 5 (N = 4)	1500
CF17_F16		Hybrid function 6 (N = 4)	1600
CF17_F17		Hybrid function 6 (N = 5)	1700
CF17_F18		Hybrid function 6 (N = 5)	1800
CF17_F19		Hybrid function 6 (N = 5)	1900
CF17_F20		Hybrid function 6 (N = 6)	2000
CF17_F21	Composition	Composition function 1 (N = 3)	2100
CF17_F22		Composition function 2 (N = 3)	2200
CF17_F23		Composition function 3 (N = 4)	2300
CF17_F24		Composition function 4 (N = 4)	2400
CF17_F25		Composition function 5 (N = 5)	2500
CF17_F26		Composition function 6 (N = 5)	2600
CF17_F27		Composition function 7 (N = 6)	2700
CF17_F28		Composition function 8 (N = 6)	2800
CF17_F29		Composition function 9 (N = 3)	2900
CF17_F30		Composition function 10 (N = 3)	3000

**Table 2:** Comparison algorithms associated with CEC2017.

Algorithms	Time	Parameters
Binary Enhanced Golden Jackal Optimization (BEGJO) [39]	2024	β=1.5, c1=1.5, E1 linearly decreasing from 1.5 to 0
DMQPSO [40]	2024	β linearly decreasing from 1 to 0.5
Fitness-Distance Balance-Based Artificial Rabbits Optimization (FDBARO) [41]	2024	No Parameters
HWEAVOA [42]	2024	$p_{1}=0.6,\;p_{2}=0.4,\;p_{3}=0.4,\;\alpha =0.8,\;\beta =0.2,\;W=2.5,\;a=1.4,\;b=0.3$
QHDBO [43]	2024	R=cos(π·(t/Tmax)+1)·0.5
Weighted Average Algorithm (WAA) [44]	2024	$f=(\alpha \cdot rand-1)\sin(\pi \cdot \frac{it}{Max_{it}})$
Human Evolutionary Optimization Algorithm (HEOA)	2024	$w=0.2\cdot \cos(\frac{\pi }{2}(1-\frac{t}{Max_{iter}}))$
Developed Human Evolutionary Optimization Algorithm (DHEOA) [45]	2025	$w=0.2\cdot \cos(\frac{\pi }{2}(1-\frac{t}{Max_{iter}}))$
Improved Human Evolution Optimization Algorithm (IHEOA) [46]	2025	$w=0.2\cdot \cos(\frac{\pi }{2}(1-\frac{t}{Max_{iter}}))$
MS-TSA [47]	2024	$z_{0}\;\mathrm{between}\;0\;\mathrm{and}\;1$
I-CPA [48]	2023	No Parameters

**Table 3:** The algorithm’s fitness function values (CEC2017) (Dim=100, MaxFEs=30,000).

Functions	Metrics	BEGJO	DMQPSO	FDBARO	HWEAVOA	QHDBO	WAA	HEOA	DHEOA	IHEOA	CLNBHEOA
CF17_F1	Mean	5.470 × 10^+04^	3.042 × 10^+03^	3.238 × 10^+08^	1.554 × 10^+03^	2.964 × 10^+03^	3.112 × 10^+03^	2.226 × 10^+03^	4.028 × 10^+04^	7.981 × 10^+05^	**1.000 × 10** ^+02^
	Std	2.823 × 10^+05^	3.299 × 10^+03^	1.432 × 10^+08^	1.981 × 10^+03^	3.317 × 10^+03^	3.214 × 10^+03^	2.134 × 10^+03^	2.036 × 10^+05^	2.707 × 10^+06^	4.594 × 10^−14^
CF17_F3	Mean	3.003 × 10^+02^	3.037 × 10^+02^	2.684 × 10^+03^	3.062 × 10^+02^	3.027 × 10^+02^	3.000 × 10^+02^	3.000 × 10^+02^	3.000 × 10^+02^	3.116 × 10^+02^	**3.000 × 10^+02^**
	Std	5.481 × 10^−01^	1.317 × 10^+01^	2.348 × 10^+03^	1.516 × 10^+01^	6.232 × 10^+00^	1.598 × 10^−09^	4.088 × 10^−10^	4.104 × 10^−02^	4.514 × 10^+01^	4.352 × 10^−14^
CF17_F4	Mean	4.126 × 10^+02^	4.067 × 10^+02^	4.336 × 10^+02^	4.024 × 10^+02^	4.072 × 10^+02^	4.078 × 10^+02^	4.034 × 10^+02^	4.095 × 10^+02^	4.309 × 10^+02^	**4.000 × 10^+02^**
	Std	1.754 × 10^+01^	1.198 × 10^+01^	2.297 × 10^+01^	1.268 × 10^+00^	1.278 × 10^+01^	1.441 × 10^+01^	1.423 × 10^+00^	1.599 × 10^+01^	4.480 × 10^+01^	2.396 × 10^−08^
CF17_F5	Mean	5.311 × 10^+02^	5.434 × 10^+02^	5.635 × 10^+02^	5.221 × 10^+02^	5.250 × 10^+02^	5.218 × 10^+02^	5.125 × 10^+02^	5.134 × 10^+02^	5.386 × 10^+02^	**5.072 × 10^+02^**
	Std	1.147 × 10^+01^	1.355 × 10^+01^	9.144 × 10^+00^	8.424 × 10^+00^	9.635 × 10^+00^	8.405 × 10^+00^	5.047 × 10^+00^	5.540 × 10^+00^	1.194 × 10^+01^	2.286 × 10^+00^
CF17_F6	Mean	6.012 × 10^+02^	6.174 × 10^+02^	6.126 × 10^+02^	6.003 × 10^+02^	6.005 × 10^+02^	6.157 × 10^+02^	6.006 × 10^+02^	6.005 × 10^+02^	6.107 × 10^+02^	**6.000 × 10^+02^**
	Std	2.533 × 10^+00^	8.682 × 10^+00^	4.898 × 10^+00^	8.496 × 10^−01^	7.983 × 10^−01^	1.253 × 10^+01^	1.459 × 10^+00^	9.855 × 10^−01^	7.438 × 10^+00^	1.198 × 10^−05^
CF17_F7	Mean	7.430 × 10^+02^	7.625 × 10^+02^	7.796 × 10^+02^	7.334 × 10^+02^	7.404 × 10^+02^	7.348 × 10^+02^	7.240 × 10^+02^	7.272 × 10^+02^	7.402 × 10^+02^	**7.176 × 10^+02^**
	Std	1.193 × 10^+01^	2.254 × 10^+01^	1.380 × 10^+01^	7.288 × 10^+00^	1.286 × 10^+01^	9.414 × 10^+00^	6.465 × 10^+00^	7.060 × 10^+00^	1.132 × 10^+01^	2.921 × 10^+00^
CF17_F8	Mean	8.184 × 10^+02^	8.336 × 10^+02^	8.434 × 10^+02^	8.130 × 10^+02^	8.269 × 10^+02^	8.281 × 10^+02^	8.106 × 10^+02^	8.182 × 10^+02^	8.310 × 10^+02^	**8.057 × 10^+02^**
	Std	4.802 × 10^+00^	8.803 × 10^+00^	1.669 × 10^+01^	3.648 × 10^+00^	1.207 × 10^+01^	1.305 × 10^+01^	4.177 × 10^+00^	7.527 × 10^+00^	1.094 × 10^+01^	2.292 × 10^+00^
CF17_F9	Mean	9.088 × 10^+02^	1.147 × 10^+03^	9.435 × 10^+02^	9.096 × 10^+02^	9.195 × 10^+02^	9.551 × 10^+02^	9.020 × 10^+02^	9.022 × 10^+02^	9.743 × 10^+02^	**9.000 × 10^+02^**
	Std	2.286 × 10^+01^	1.982 × 10^+02^	3.116 × 10^+01^	3.055 × 10^+01^	4.565 × 10^+01^	1.045 × 10^+02^	3.980 × 10^+00^	4.838 × 10^+00^	1.139 × 10^+02^	0.000 × 10^+00^
CF17_F10	Mean	1.554 × 10^+03^	1.870 × 10^+03^	2.581 × 10^+03^	1.639 × 10^+03^	1.852 × 10^+03^	1.898 × 10^+03^	1.986 × 10^+03^	1.734 × 10^+03^	1.995 × 10^+03^	**1.299 × 10^+03^**
	Std	2.869 × 10^+02^	3.439 × 10^+02^	4.125 × 10^+02^	1.752 × 10^+02^	2.865 × 10^+02^	2.944 × 10^+02^	2.801 × 10^+02^	3.176 × 10^+02^	3.067 × 10^+02^	1.419 × 10^+02^
CF17_F11	Mean	1.124 × 10^+03^	1.159 × 10^+03^	1.213 × 10^+03^	1.109 × 10^+03^	1.114 × 10^+03^	1.162 × 10^+03^	1.118 × 10^+03^	1.128 × 10^+03^	1.227 × 10^+03^	**1.101 × 10^+03^**
	Std	1.006 × 10^+01^	5.745 × 10^+01^	9.193 × 10^+01^	5.848 × 10^+00^	1.267 × 10^+01^	4.330 × 10^+01^	1.898 × 10^+01^	2.979 × 10^+01^	1.120 × 10^+02^	1.298 × 10^+00^
CF17_F12	Mean	7.460 × 10^+03^	6.561 × 10^+05^	2.192 × 10^+07^	9.042 × 10^+03^	1.829 × 10^+06^	2.340 × 10^+06^	1.667 × 10^+04^	2.972 × 10^+04^	1.908 × 10^+06^	**1.321 × 10^+03^**
	Std	2.941 × 10^+03^	8.157 × 10^+05^	1.941 × 10^+07^	4.121 × 10^+03^	3.708 × 10^+06^	1.941 × 10^+06^	1.596 × 10^+04^	8.597 × 10^+04^	3.230 × 10^+06^	9.833 × 10^+01^
CF17_F13	Mean	9.174 × 10^+03^	1.243 × 10^+04^	1.792 × 10^+06^	3.738 × 10^+03^	9.062 × 10^+03^	1.461 × 10^+04^	7.015 × 10^+03^	8.635 × 10^+03^	1.174 × 10^+04^	**1.307 × 10^+03^**
	Std	3.531 × 10^+03^	9.746 × 10^+03^	2.050 × 10^+06^	2.101 × 10^+03^	7.772 × 10^+03^	1.274 × 10^+04^	5.612 × 10^+03^	6.017 × 10^+03^	1.151 × 10^+04^	3.204 × 10^+00^
CF17_F14	Mean	1.488 × 10^+03^	2.170 × 10^+03^	2.813 × 10^+05^	1.504 × 10^+03^	1.527 × 10^+03^	1.645 × 10^+03^	1.478 × 10^+03^	1.466 × 10^+03^	1.719 × 10^+03^	**1.402 × 10^+03^**
	Std	3.098 × 10^+01^	9.216 × 10^+02^	5.167 × 10^+05^	2.896 × 10^+01^	6.726 × 10^+01^	3.296 × 10^+02^	2.411 × 10^+01^	6.426 × 10^+01^	4.939 × 10^+02^	1.071 × 10^+00^
CF17_F15	Mean	2.040 × 10^+03^	4.224 × 10^+03^	3.891 × 10^+04^	1.819 × 10^+03^	2.142 × 10^+03^	3.961 × 10^+03^	1.704 × 10^+03^	2.519 × 10^+03^	2.417 × 10^+03^	**1.501 × 10^+03^**
	Std	9.606 × 10^+02^	2.692 × 10^+03^	6.415 × 10^+04^	2.698 × 10^+02^	8.484 × 10^+02^	2.675 × 10^+03^	9.180 × 10^+01^	1.076 × 10^+03^	9.134 × 10^+02^	1.072 × 10^+00^
CF17_F16	Mean	1.748 × 10^+03^	1.763 × 10^+03^	1.870 × 10^+03^	1.679 × 10^+03^	1.742 × 10^+03^	1.755 × 10^+03^	1.623 × 10^+03^	1.709 × 10^+03^	1.754 × 10^+03^	**1.613 × 10^+03^**
	Std	1.495 × 10^+02^	1.221 × 10^+02^	1.415 × 10^+02^	6.130 × 10^+01^	1.053 × 10^+02^	1.119 × 10^+02^	4.034 × 10^+01^	8.813 × 10^+01^	1.212 × 10^+02^	3.112 × 10^+01^
CF17_F17	Mean	1.768 × 10^+03^	1.767 × 10^+03^	1.827 × 10^+03^	1.742 × 10^+03^	1.782 × 10^+03^	1.771 × 10^+03^	1.741 × 10^+03^	1.742 × 10^+03^	1.769 × 10^+03^	**1.703 × 10^+03^**
	Std	3.236 × 10^+01^	3.434 × 10^+01^	5.324 × 10^+01^	7.993 × 10^+00^	3.992 × 10^+01^	2.714 × 10^+01^	1.266 × 10^+01^	2.539 × 10^+01^	3.142 × 10^+01^	4.960 × 10^+00^
CF17_F18	Mean	5.919 × 10^+03^	1.516 × 10^+04^	8.325 × 10^+06^	2.971 × 10^+03^	1.052 × 10^+04^	2.042 × 10^+04^	8.224 × 10^+03^	1.306 × 10^+04^	1.878 × 10^+04^	**1.805 × 10^+03^**
	Std	5.329 × 10^+03^	1.214 × 10^+04^	1.013 × 10^+07^	1.177 × 10^+03^	8.408 × 10^+03^	1.230 × 10^+04^	5.657 × 10^+03^	1.063 × 10^+04^	1.606 × 10^+04^	7.734 × 10^+00^
CF17_F19	Mean	1.956 × 10^+03^	9.568 × 10^+03^	8.819 × 10^+05^	2.216 × 10^+03^	3.366 × 10^+03^	4.883 × 10^+03^	2.028 × 10^+03^	8.424 × 10^+03^	5.161 × 10^+03^	**1.900 × 10^+03^**
	Std	4.675 × 10^+01^	9.040 × 10^+03^	1.899 × 10^+06^	5.089 × 10^+02^	2.112 × 10^+03^	3.225 × 10^+03^	1.052 × 10^+02^	6.840 × 10^+03^	6.896 × 10^+03^	3.722 × 10^−01^
CF17_F20	Mean	2.046 × 10^+03^	2.133 × 10^+03^	2.185 × 10^+03^	2.032 × 10^+03^	2.061 × 10^+03^	2.114 × 10^+03^	2.022 × 10^+03^	2.092 × 10^+03^	2.097 × 10^+03^	**2.001 × 10^+03^**
	Std	1.831 × 10^+01^	7.504 × 10^+01^	6.224 × 10^+01^	1.590 × 10^+01^	4.496 × 10^+01^	6.861 × 10^+01^	1.060 × 10^+01^	5.782 × 10^+01^	5.656 × 10^+01^	1.151 × 10^+00^
CF17_F21	Mean	2.314 × 10^+03^	2.271 × 10^+03^	2.350 × 10^+03^	2.256 × 10^+03^	2.322 × 10^+03^	2.283 × 10^+03^	2.277 × 10^+03^	2.286 × 10^+03^	**2.232 × 10^+03^**	2.248 × 10^+03^
	Std	4.398 × 10^+01^	6.876 × 10^+01^	2.830 × 10^+01^	5.517 × 10^+01^	2.455 × 10^+01^	5.881 × 10^+01^	4.913 × 10^+01^	4.787 × 10^+01^	5.263 × 10^+01^	5.603 × 10^+01^
CF17_F22	Mean	**2.272 × 10^+03^**	2.308 × 10^+03^	2.338 × 10^+03^	2.300 × 10^+03^	2.303 × 10^+03^	2.297 × 10^+03^	2.297 × 10^+03^	2.300 × 10^+03^	2.310 × 10^+03^	2.294 × 10^+03^
	Std	4.078 × 10^+01^	4.584 × 10^+00^	1.613 × 10^+01^	1.415 × 10^+00^	2.244 × 10^+00^	2.359 × 10^+01^	1.946 × 10^+01^	1.171 × 10^+01^	7.111 × 10^+00^	2.406 × 10^+01^
CF17_F23	Mean	2.644 × 10^+03^	2.645 × 10^+03^	2.668 × 10^+03^	2.613 × 10^+03^	2.630 × 10^+03^	2.621 × 10^+03^	2.615 × 10^+03^	2.619 × 10^+03^	2.642 × 10^+03^	**2.610 × 10^+03^**
	Std	1.779 × 10^+01^	1.876 × 10^+01^	8.838 × 10^+00^	5.935 × 10^+00^	1.528 × 10^+01^	8.681 × 10^+00^	5.536 × 10^+00^	8.574 × 10^+00^	1.349 × 10^+01^	2.528 × 10^+00^
CF17_F24	Mean	2.737 × 10^+03^	2.765 × 10^+03^	2.800 × 10^+03^	2.718 × 10^+03^	2.758 × 10^+03^	2.741 × 10^+03^	2.704 × 10^+03^	2.744 × 10^+03^	2.671 × 10^+03^	**2.644 × 10^+03^**
	Std	1.101 × 10^+02^	7.559 × 10^+01^	1.272 × 10^+01^	6.500 × 10^+01^	1.164 × 10^+01^	4.663 × 10^+01^	8.007 × 10^+01^	8.141 × 10^+00^	1.157 × 10^+02^	1.196 × 10^+02^
CF17_F25	Mean	2.922 × 10^+03^	2.937 × 10^+03^	2.950 × 10^+03^	2.927 × 10^+03^	2.923 × 10^+03^	2.917 × 10^+03^	2.924 × 10^+03^	2.935 × 10^+03^	2.941 × 10^+03^	**2.912 × 10^+03^**
	Std	2.271 × 10^+01^	2.824 × 10^+01^	2.121 × 10^+01^	2.090 × 10^+01^	2.375 × 10^+01^	6.672 × 10^+01^	2.334 × 10^+01^	2.052 × 10^+01^	2.637 × 10^+01^	2.112 × 10^+01^
CF17_F26	Mean	2.947 × 10^+03^	3.118 × 10^+03^	3.266 × 10^+03^	2.907 × 10^+03^	3.215 × 10^+03^	2.965 × 10^+03^	2.941 × 10^+03^	3.010 × 10^+03^	3.023 × 10^+03^	**2.905 × 10^+03^**
	Std	2.533 × 10^+02^	3.427 × 10^+02^	4.882 × 10^+02^	6.786 × 10^+01^	3.558 × 10^+02^	2.843 × 10^+02^	8.060 × 10^+01^	2.247 × 10^+02^	1.445 × 10^+02^	1.798 × 10^+01^
CF17_F27	Mean	3.105 × 10^+03^	3.108 × 10^+03^	3.109 × 10^+03^	3.092 × 10^+03^	3.110 × 10^+03^	3.098 × 10^+03^	3.093 × 10^+03^	3.097 × 10^+03^	3.105 × 10^+03^	**3.090 × 10^+03^**
	Std	8.564 × 10^+00^	2.311 × 10^+01^	1.684 × 10^+01^	2.763 × 10^+00^	1.199 × 10^+01^	1.791 × 10^+01^	3.020 × 10^+00^	1.173 × 10^+01^	1.850 × 10^+01^	9.345 × 10^−01^
CF17_F28	Mean	3.459 × 10^+03^	3.308 × 10^+03^	3.381 × 10^+03^	**3.209 × 10^+03^**	3.328 × 10^+03^	3.273 × 10^+03^	3.267 × 10^+03^	3.337 × 10^+03^	3.300 × 10^+03^	3.230 × 10^+03^
	Std	1.364 × 10^+02^	1.528 × 10^+02^	1.103 × 10^+02^	1.320 × 10^+02^	1.181 × 10^+02^	1.474 × 10^+02^	1.362 × 10^+02^	9.853 × 10^+01^	1.018 × 10^+02^	1.509 × 10^+02^
CF17_F29	Mean	3.206 × 10^+03^	3.286 × 10^+03^	3.336 × 10^+03^	3.193 × 10^+03^	3.232 × 10^+03^	3.211 × 10^+03^	3.181 × 10^+03^	3.202 × 10^+03^	3.276 × 10^+03^	**3.152 × 10^+03^**
	Std	2.861 × 10^+01^	7.563 × 10^+01^	5.761 × 10^+01^	2.078 × 10^+01^	6.186 × 10^+01^	4.800 × 10^+01^	1.967 × 10^+01^	4.596 × 10^+01^	6.796 × 10^+01^	1.063 × 10^+01^
CF17_F30	Mean	9.854 × 10^+04^	3.599 × 10^+05^	2.222 × 10^+06^	9.285 × 10^+04^	3.151 × 10^+05^	4.437 × 10^+05^	3.566 × 10^+05^	2.669 × 10^+05^	6.622 × 10^+05^	**3.514 × 10^+03^**
	Std	2.735 × 10^+05^	4.322 × 10^+05^	3.032 × 10^+06^	2.059 × 10^+05^	4.422 × 10^+05^	6.321 × 10^+05^	4.681 × 10^+05^	4.822 × 10^+05^	7.298 × 10^+05^	8.863 × 10^+01^
Mean Rank		5.14	7.69	9.72	3.24	6.07	6.34	3.21	5.03	7.31	**1.10**
Final Rank		5	9	10	3	6	7	2	4	8	**1**

**Table 4:** The algorithm’s fitness function values (CEC2017) (Dim=30,MaxFEs=300,000).

Functions	Metrics	MS-TSA	I-CPA	FDBARO	HWEAVOA	QHDBO	WAA	HEOA	DHEOA	IHEOA	CLNBHEOA
CF17_F1	Mean	4.370 × 10^+03^	6.580 × 10^+09^	3.853 × 10^+08^	1.792 × 10^+03^	2.230 × 10^+03^	1.711 × 10^+03^	1.350 × 10^+03^	2.277 × 10^+03^	6.436 × 10^+03^	**1.000 × 10^+02^**
	Std	5.600 × 10^+03^	4.510 × 10^+09^	3.200 × 10^+08^	1.923 × 10^+03^	2.534 × 10^+03^	2.142 × 10^+03^	1.606 × 10^+03^	2.541 × 10^+03^	4.242 × 10^+03^	0.000 × 10^+00^
CF17_F3	Mean	8.120 × 10^+03^	5.220 × 10^+04^	2.396 × 10^+03^	**3.000 × 10^+02^**	**3.000 × 10^+02^**	**3.000 × 10^+02^**	**3.000 × 10^+02^**	**3.000 × 10^+02^**	**3.000 × 10^+02^**	**3.000 × 10^+02^**
	Std	3.150 × 10^+03^	8.930 × 10^+03^	2.063 × 10^+03^	1.007 × 10^−13^	6.064 × 10^−14^	3.309 × 10^−10^	8.039 × 10^−14^	1.185 × 10^−13^	2.728 × 10^−13^	0.000 × 10^+00^
CF17_F4	Mean	4.530 × 10^+02^	1.840 × 10^+03^	4.272 × 10^+02^	4.001 × 10^+02^	4.028 × 10^+02^	4.001 × 10^+02^	**4.000 × 10^+02^**	4.003 × 10^+02^	4.104 × 10^+02^	**4.000 × 10^+02^**
	Std	3.390 × 10^+01^	1.090 × 10^+03^	1.720 × 10^+01^	2.526 × 10^−02^	1.884 × 10^+00^	2.380 × 10^−01^	2.060 × 10^−02^	1.440 × 10^−01^	1.528 × 10^+01^	0.000 × 10^+00^
CF17_F5	Mean	6.240 × 10^+02^	7.280 × 10^+02^	5.538 × 10^+02^	5.147 × 10^+02^	5.224 × 10^+02^	5.159 × 10^+02^	5.100 × 10^+02^	5.136 × 10^+02^	5.267 × 10^+02^	**5.066 × 10^+02^**
	Std	6.570 × 10^+01^	4.900 × 10^+01^	1.029 × 10^+01^	6.363 × 10^+00^	8.643 × 10^+00^	6.316 × 10^+00^	3.606 × 10^+00^	6.456 × 10^+00^	1.244 × 10^+01^	2.283 × 10^+00^
CF17_F6	Mean	**6.000 × 10^+02^**	6.420 × 10^+02^	6.115 × 10^+02^	6.001 × 10^+02^	6.003 × 10^+02^	6.018 × 10^+02^	6.003 × 10^+02^	6.002 × 10^+02^	6.073 × 10^+02^	**6.000 × 10^+02^**
	Std	2.350 × 10^−01^	7.320 × 10^+00^	2.965 × 10^+00^	2.009 × 10^−01^	4.866 × 10^−01^	2.426 × 10^+00^	7.679 × 10^−01^	5.185 × 10^−01^	6.315 × 10^+00^	2.111 × 10^−14^
CF17_F7	Mean	8.960 × 10^+02^	1.100 × 10^+03^	7.770 × 10^+02^	7.300 × 10^+02^	7.256 × 10^+02^	7.279 × 10^+02^	7.190 × 10^+02^	7.259 × 10^+02^	7.439 × 10^+02^	**7.182 × 10^+02^**
	Std	5.010 × 10^+01^	8.140 × 10^+01^	1.099 × 10^+01^	7.209 × 10^+00^	9.149 × 10^+00^	8.671 × 10^+00^	4.072 × 10^+00^	6.837 × 10^+00^	1.197 × 10^+01^	3.557 × 10^+00^
CF17_F8	Mean	9.280 × 10^+02^	9.930 × 10^+02^	8.366 × 10^+02^	8.090 × 10^+02^	8.213 × 10^+02^	8.159 × 10^+02^	8.093 × 10^+02^	8.131 × 10^+02^	8.284 × 10^+02^	**8.053 × 10^+02^**
	Std	6.630 × 10^+01^	3.510 × 10^+01^	9.495 × 10^+00^	2.629 × 10^+00^	6.345 × 10^+00^	6.868 × 10^+00^	3.108 × 10^+00^	4.638 × 10^+00^	8.632 × 10^+00^	1.597 × 10^+00^
CF17_F9	Mean	9.010 × 10^+02^	5.470 × 10^+03^	9.486 × 10^+02^	9.002 × 10^+02^	9.020 × 10^+02^	9.011 × 10^+02^	9.015 × 10^+02^	9.017 × 10^+02^	9.436 × 10^+02^	**9.000 × 10^+02^**
	Std	1.140 × 10^+00^	1.600 × 10^+03^	4.633 × 10^+01^	4.684 × 10^−01^	5.830 × 10^+00^	2.234 × 10^+00^	2.265 × 10^+00^	8.098 × 10^+00^	6.363 × 10^+01^	0.000 × 10^+00^
CF17_F10	Mean	7.100 × 10^+03^	6.000 × 10^+03^	2.377 × 10^+03^	1.435 × 10^+03^	1.745 × 10^+03^	1.645 × 10^+03^	1.304 × 10^+03^	1.591 × 10^+03^	1.790 × 10^+03^	**1.279 × 10^+03^**
	Std	1.530 × 10^+03^	6.680 × 10^+02^	4.389 × 10^+02^	1.395 × 10^+02^	2.904 × 10^+02^	2.182 × 10^+02^	1.986 × 10^+02^	2.558 × 10^+02^	2.855 × 10^+02^	1.794 × 10^+02^
CF17_F11	Mean	1.170 × 10^+03^	3.490 × 10^+03^	1.181 × 10^+03^	1.104 × 10^+03^	1.112 × 10^+03^	1.133 × 10^+03^	1.111 × 10^+03^	1.116 × 10^+03^	1.173 × 10^+03^	**1.101 × 10^+03^**
	Std	4.890 × 10^+01^	1.810 × 10^+03^	5.112 × 10^+01^	3.404 × 10^+00^	8.111 × 10^+00^	2.325 × 10^+01^	1.009 × 10^+01^	8.447 × 10^+00^	6.107 × 10^+01^	1.207 × 10^+00^
CF17_F12	Mean	2.060 × 10^+05^	6.370 × 10^+08^	2.275 × 10^+07^	6.805 × 10^+03^	3.021 × 10^+05^	1.957 × 10^+04^	1.079 × 10^+04^	1.188 × 10^+04^	5.464 × 10^+05^	**1.294 × 10^+03^**
	Std	1.140 × 10^+05^	7.140 × 10^+08^	3.035 × 10^+07^	2.654 × 10^+03^	6.095 × 10^+05^	1.824 × 10^+04^	1.040 × 10^+04^	1.091 × 10^+04^	1.796 × 10^+06^	9.653 × 10^+01^
CF17_F13	Mean	1.480 × 10^+04^	6.370 × 10^+08^	1.155 × 10^+06^	2.416 × 10^+03^	2.522 × 10^+03^	1.052 × 10^+04^	1.949 × 10^+03^	7.999 × 10^+03^	8.345 × 10^+03^	**1.307 × 10^+03^**
	Std	1.480 × 10^+04^	1.320 × 10^+09^	1.439 × 10^+06^	8.483 × 10^+02^	1.673 × 10^+03^	8.300 × 10^+03^	6.000 × 10^+02^	6.887 × 10^+03^	9.312 × 10^+03^	3.127 × 10^+00^
CF17_F14	Mean	7.180 × 10^+03^	1.020 × 10^+06^	2.927 × 10^+05^	1.423 × 10^+03^	1.451 × 10^+03^	1.456 × 10^+03^	1.425 × 10^+03^	1.426 × 10^+03^	1.486 × 10^+03^	**1.402 × 10^+03^**
	Std	4.330 × 10^+03^	7.340 × 10^+05^	4.628 × 10^+05^	6.130 × 10^+00^	2.465 × 10^+01^	2.469 × 10^+01^	1.196 × 10^+01^	1.314 × 10^+01^	4.387 × 10^+01^	7.221 × 10^−01^
CF17_F15	Mean	6.870 × 10^+03^	7.210 × 10^+06^	6.155 × 10^+04^	1.513 × 10^+03^	1.541 × 10^+03^	1.619 × 10^+03^	1.513 × 10^+03^	1.524 × 10^+03^	1.659 × 10^+03^	**1.501 × 10^+03^**
	Std	7.010 × 10^+03^	1.950 × 10^+07^	9.634 × 10^+04^	6.968 × 10^+00^	3.751 × 10^+01^	5.685 × 10^+01^	1.390 × 10^+01^	1.894 × 10^+01^	9.289 × 10^+01^	7.846 × 10^−01^
CF17_F16	Mean	2.430 × 10^+03^	3.230 × 10^+03^	1.857 × 10^+03^	1.610 × 10^+03^	1.658 × 10^+03^	1.646 × 10^+03^	1.623 × 10^+03^	1.680 × 10^+03^	1.697 × 10^+03^	**1.608 × 10^+03^**
	Std	4.550 × 10^+02^	3.970 × 10^+02^	1.733 × 10^+02^	2.898 × 10^+01^	6.980 × 10^+01^	5.531 × 10^+01^	4.515 × 10^+01^	7.447 × 10^+01^	9.699 × 10^+01^	2.318 × 10^+01^
CF17_F17	Mean	1.950 × 10^+03^	2.310 × 10^+03^	1.826 × 10^+03^	1.732 × 10^+03^	1.752 × 10^+03^	1.747 × 10^+03^	1.733 × 10^+03^	1.726 × 10^+03^	1.749 × 10^+03^	**1.703 × 10^+03^**
	Std	1.750 × 10^+02^	2.160 × 10^+02^	5.685 × 10^+01^	9.655 × 10^+00^	4.067 × 10^+01^	2.250 × 10^+01^	1.003 × 10^+01^	1.825 × 10^+01^	1.433 × 10^+01^	4.944 × 10^+00^
CF17_F18	Mean	5.710 × 10^+05^	4.360 × 10^+06^	5.424 × 10^+06^	2.856 × 10^+03^	1.006 × 10^+04^	1.194 × 10^+04^	4.412 × 10^+03^	1.577 × 10^+04^	1.659 × 10^+04^	**1.802 × 10^+03^**
	Std	5.220 × 10^+05^	5.170 × 10^+06^	4.735 × 10^+06^	1.057 × 10^+03^	1.204 × 10^+04^	9.162 × 10^+03^	2.557 × 10^+03^	1.077 × 10^+04^	1.711 × 10^+04^	4.795 × 10^+00^
CF17_F19	Mean	8.260 × 10^+03^	2.050 × 10^+07^	8.285 × 10^+05^	1.909 × 10^+03^	1.926 × 10^+03^	1.936 × 10^+03^	1.906 × 10^+03^	2.318 × 10^+03^	2.082 × 10^+03^	**1.900 × 10^+03^**
	Std	8.410 × 10^+03^	3.680 × 10^+07^	1.619 × 10^+06^	3.241 × 10^+00^	3.012 × 10^+01^	2.384 × 10^+01^	2.628 × 10^+00^	1.085 × 10^+03^	2.712 × 10^+02^	4.137 × 10^−01^
CF17_F20	Mean	2.230 × 10^+03^	2.650 × 10^+03^	2.162 × 10^+03^	2.016 × 10^+03^	2.041 × 10^+03^	2.044 × 10^+03^	2.021 × 10^+03^	2.037 × 10^+03^	2.078 × 10^+03^	**2.000 × 10^+03^**
	Std	1.500 × 10^+02^	1.590 × 10^+02^	5.858 × 10^+01^	1.087 × 10^+01^	4.659 × 10^+01^	2.867 × 10^+01^	1.132 × 10^+01^	4.000 × 10^+01^	5.295 × 10^+01^	5.700 × 10^−02^
CF17_F21	Mean	2.410 × 10^+03^	2.510 × 10^+03^	2.333 × 10^+03^	2.249 × 10^+03^	2.323 × 10^+03^	2.239 × 10^+03^	2.252 × 10^+03^	2.295 × 10^+03^	2.252 × 10^+03^	**2.204 × 10^+03^**
	Std	6.270 × 10^+01^	3.420 × 10^+01^	4.335 × 10^+01^	5.676 × 10^+01^	2.471 × 10^+01^	6.097 × 10^+01^	5.538 × 10^+01^	4.315 × 10^+01^	5.627 × 10^+01^	1.087 × 10^+00^
CF17_F22	Mean	2.880 × 10^+03^	5.240 × 10^+03^	2.332 × 10^+03^	2.300 × 10^+03^	2.333 × 10^+03^	2.305 × 10^+03^	2.296 × 10^+03^	2.298 × 10^+03^	2.309 × 10^+03^	**2.292 × 10^+03^**
	Std	1.620 × 10^+03^	2.210 × 10^+03^	7.154 × 10^+00^	3.050 × 10^−01^	1.574 × 10^+02^	2.188 × 10^+00^	2.059 × 10^+01^	1.506 × 10^+01^	7.373 × 10^+00^	2.510 × 10^+01^
CF17_F23	Mean	2.730 × 10^+03^	3.080 × 10^+03^	2.666 × 10^+03^	2.612 × 10^+03^	2.623 × 10^+03^	2.617 × 10^+03^	2.614 × 10^+03^	2.615 × 10^+03^	2.635 × 10^+03^	**2.610 × 10^+03^**
	Std	4.560 × 10^+01^	1.220 × 10^+02^	1.011 × 10^+01^	4.234 × 10^+00^	8.796 × 10^+00^	6.911 × 10^+00^	5.506 × 10^+00^	6.909 × 10^+00^	1.336 × 10^+01^	3.366 × 10^+00^
CF17_F24	Mean	2.980 × 10^+03^	3.220 × 10^+03^	2.789 × 10^+03^	2.706 × 10^+03^	2.749 × 10^+03^	2.742 × 10^+03^	2.732 × 10^+03^	2.744 × 10^+03^	**2.557 × 10^+03^**	2.635 × 10^+03^
	Std	5.370 × 10^+01^	8.790 × 10^+01^	5.087 × 10^+01^	8.217 × 10^+01^	4.814 × 10^+01^	1.041 × 10^+01^	4.421 × 10^+01^	5.488 × 10^+00^	1.090 × 10^+02^	1.205 × 10^+02^
CF17_F25	Mean	**2.890 × 10^+03^**	3.170 × 10^+03^	2.949 × 10^+03^	2.921 × 10^+03^	2.923 × 10^+03^	2.920 × 10^+03^	2.925 × 10^+03^	2.937 × 10^+03^	2.931 × 10^+03^	2.915 × 10^+03^
	Std	8.590 × 10^+00^	1.180 × 10^+02^	2.167 × 10^+01^	2.292 × 10^+01^	2.332 × 10^+01^	2.323 × 10^+01^	2.312 × 10^+01^	1.959 × 10^+01^	2.596 × 10^+01^	2.220 × 10^+01^
CF17_F26	Mean	4.620 × 10^+03^	7.520 × 10^+03^	3.180 × 10^+03^	**2.875 × 10^+03^**	3.264 × 10^+03^	2.905 × 10^+03^	2.954 × 10^+03^	2.937 × 10^+03^	3.038 × 10^+03^	2.882 × 10^+03^
	Std	6.550 × 10^+02^	1.060 × 10^+03^	4.030 × 10^+02^	8.561 × 10^+01^	4.072 × 10^+02^	1.514 × 10^+01^	1.291 × 10^+02^	1.221 × 10^+02^	1.112 × 10^+02^	7.702 × 10^+01^
CF17_F27	Mean	3.220 × 10^+03^	3.590 × 10^+03^	3.106 × 10^+03^	3.091 × 10^+03^	3.106 × 10^+03^	3.091 × 10^+03^	3.095 × 10^+03^	3.097 × 10^+03^	3.097 × 10^+03^	**3.090 × 10^+03^**
	Std	1.360 × 10^+01^	1.430 × 10^+02^	6.123 × 10^+00^	1.784 × 10^+00^	1.077 × 10^+01^	2.094 × 10^+00^	3.625 × 10^+00^	8.231 × 10^+00^	4.143 × 10^+00^	6.596 × 10^−01^
CF17_F28	Mean	3.140 × 10^+03^	3.860 × 10^+03^	3.358 × 10^+03^	3.206 × 10^+03^	3.300 × 10^+03^	3.180 × 10^+03^	3.254 × 10^+03^	3.197 × 10^+03^	3.224 × 10^+03^	**3.100 × 10^+03^**
	Std	5.960 × 10^+01^	2.880 × 10^+02^	8.745 × 10^+01^	1.406 × 10^+02^	1.360 × 10^+02^	1.352 × 10^+02^	1.419 × 10^+02^	7.235 × 10^+01^	9.211 × 10^+01^	1.259 × 10^−05^
CF17_F29	Mean	3.510 × 10^+03^	4.720 × 10^+03^	3.285 × 10^+03^	3.164 × 10^+03^	3.192 × 10^+03^	3.162 × 10^+03^	3.160 × 10^+03^	3.202 × 10^+03^	3.197 × 10^+03^	**3.143 × 10^+03^**
	Std	1.820 × 10^+02^	4.080 × 10^+02^	6.727 × 10^+01^	1.743 × 10^+01^	3.464 × 10^+01^	1.973 × 10^+01^	1.875 × 10^+01^	4.422 × 10^+01^	3.806 × 10^+01^	8.508 × 10^+00^
CF17_F30	Mean	8.500 × 10^+03^	4.530 × 10^+07^	1.427 × 10^+06^	6.665 × 10^+03^	9.272 × 10^+04^	7.678 × 10^+04^	2.308 × 10^+05^	6.174 × 10^+04^	4.548 × 10^+05^	**3.431 × 10^+03^**
	Std	3.640 × 10^+03^	8.510 × 10^+07^	1.258 × 10^+06^	2.995 × 10^+03^	2.472 × 10^+05^	2.679 × 10^+05^	4.560 × 10^+05^	2.064 × 10^+05^	8.935 × 10^+05^	6.031 × 10^+01^
Mean Rank		7.41	9.93	8.41	2.83	5.59	4.52	3.38	4.66	6.41	**1.10**
Final Rank		8	10	9	2	6	4	3	5	7	**1**

**Table 5:** The algorithm’s Wilcoxon rank-sum test results (CEC2017).

Functions	BEGJO	DMQPSO	FDBARO	HWEAVOA	QHDBO	WAA	HEOA	DHEOA	IHEOA
CF17_F1	1.212 × 10^−12/^−	1.212 × 10^−12/^−	1.212 × 10^−12/^−	1.212 × 10^−12/^−	1.212 × 10^−12/^−	1.212 × 10^−12/^−	1.212 × 10^−12/^−	1.212 × 10^−12/^−	1.158 × 10^−12/^−
CF17_F3	1.212 × 10^−12/^−	1.212 × 10^−12/^−	1.212 × 10^−12/^−	1.212 × 10^−12/^−	1.212 × 10^−12/^−	1.212 × 10^−12/^−	1.271 × 10^−05/^−	1.212 × 10^−12/^−	1.212 × 10^−12/^−
CF17_F4	2.705 × 10^−11/^−	2.705 × 10^−11/^−	2.705 × 10^−11/^−	2.705 × 10^−11/^−	2.705 × 10^−11/^−	2.705 × 10^−11/^−	2.705 × 10^−11/^−	2.705 × 10^−11/^−	2.688 × 10^−11/^−
CF17_F5	3.897 × 10^−11/^−	2.885 × 10^−11/^−	2.885 × 10^−11/^−	1.047 × 10^−10/^−	7.070 × 10^−11/^−	6.406 × 10^−11/^−	2.813 × 10^−06/^−	2.653 × 10^−06/^−	2.885 × 10^−11/^−
CF17_F6	2.559 × 10^−11/^−	2.559 × 10^−11/^−	2.559 × 10^−11/^−	3.132 × 10^−11/^−	2.559 × 10^−11/^−	2.559 × 10^−11/^−	3.829 × 10^−11/^−	2.559 × 10^−11/^−	2.559 × 10^−11/^−
CF17_F7	3.338 × 10^−11/^−	3.020 × 10^−11/^−	3.020 × 10^−11/^−	5.494 × 10^−11/^−	4.077 × 10^−11/^−	4.200 × 10^−10/^−	1.996 × 10^−05/^−	7.088 × 10^−08/^−	5.573 × 10^−10/^−
CF17_F8	2.894 × 10^−11/^−	2.894 × 10^−11/^−	2.894 × 10^−11/^−	4.445 × 10^−10/^−	1.551 × 10^−10/^−	2.894 × 10^−11/^−	4.334 × 10^−07/^−	1.531 × 10^−10/^−	2.894 × 10^−11/^−
CF17_F9	1.212 × 10^−12/^−	1.212 × 10^−12/^−	1.212 × 10^−12/^−	1.212 × 10^−12/^−	1.212 × 10^−12/^−	1.212 × 10^−12/^−	1.641 × 10^−11/^−	1.212 × 10^−12/^−	4.574 × 10^−12/^−
CF17_F10	3.770 × 10^−04/^−	1.070 × 10^−09/^−	3.020 × 10^−11/^−	3.197 × 10^−09/^−	7.119 × 10^−09/^−	8.993 × 10^−11/^−	8.101 × 10^−10/^−	3.804 × 10^−07/^−	4.778 × 10^−09/^−
CF17_F11	2.644 × 10^−11/^−	2.389 × 10^−11/^−	2.389 × 10^−11/^−	1.067 × 10^−10/^−	5.340 × 10^−11/^−	2.389 × 10^−11/^−	3.956 × 10^−11/^−	2.925 × 10^−11/^−	2.389 × 10^−11/^−
CF17_F12	3.020 × 10^−11/^−	3.020 × 10^−11/^−	3.020 × 10^−11/^−	3.020 × 10^−11/^−	3.020 × 10^−11/^−	3.020 × 10^−11/^−	3.020 × 10^−11/^−	3.020 × 10^−11/^−	3.020 × 10^−11/^−
CF17_F13	3.018 × 10^−11/^−	3.018 × 10^−11/^−	3.018 × 10^−11/^−	3.018 × 10^−11/^−	3.018 × 10^−11/^−	3.018 × 10^−11/^−	3.018 × 10^−11/^−	3.018 × 10^−11/^−	3.018 × 10^−11/^−
CF17_F14	2.879 × 10^−11/^−	2.879 × 10^−11/^−	2.879 × 10^−11/^−	2.879 × 10^−11/^−	2.879 × 10^−11/^−	2.879 × 10^−11/^−	2.879 × 10^−11/^−	2.879 × 10^−11/^−	2.879 × 10^−11/^−
CF17_F15	3.020 × 10^−11/^−	3.020 × 10^−11/^−	3.020 × 10^−11/^−	3.020 × 10^−11/^−	3.020 × 10^−11/^−	3.020 × 10^−11/^−	3.020 × 10^−11/^−	3.338 × 10^−11/^−	3.020 × 10^−11/^−
CF17_F16	1.010 × 10^−08/^−	8.891 × 10^−10/^−	5.494 × 10^−11/^−	3.646 × 10^−08/^−	2.227 × 10^−09/^−	7.380 × 10^−10/^−	3.368 × 10^−05/^−	1.698 × 10^−08/^−	1.411 × 10^−09/^−
CF17_F17	3.014 × 10^−11/^−	3.014 × 10^−11/^−	3.014 × 10^−11/^−	3.014 × 10^−11/^−	3.014 × 10^−11/^−	3.014 × 10^−11/^−	3.014 × 10^−11/^−	7.376 × 10^−11/^−	1.462 × 10^−10/^−
CF17_F18	3.020 × 10^−11/^−	3.020 × 10^−11/^−	3.020 × 10^−11/^−	3.020 × 10^−11/^−	3.020 × 10^−11/^−	3.020 × 10^−11/^−	3.020 × 10^−11/^−	3.020 × 10^−11/^−	3.020 × 10^−11/^−
CF17_F19	2.951 × 10^−11/^−	2.951 × 10^−11/^−	2.951 × 10^−11/^−	2.951 × 10^−11/^−	2.951 × 10^−11/^−	2.951 × 10^−11/^−	2.951 × 10^−11/^−	2.951 × 10^−11/^−	2.951 × 10^−11/^−
CF17_F20	1.263 × 10^−11/^−	1.263 × 10^−11/^−	1.263 × 10^−11/^−	1.263 × 10^−11/^−	1.555 × 10^−11/^−	1.263 × 10^−11/^−	2.351 × 10^−11/^−	1.913 × 10^−11/^−	1.263 × 10^−11/^−
CF17_F21	2.772 × 10^−09/^−	1.412 × 10^−04/^−	6.673 × 10^−11/^−	9.468 × 10^−03/^−	1.100 × 10^−09/^−	8.623 × 10^−06/^−	7.943 × 10^−03/^−	5.666 × 10^−04/^−	6.262 × 10^−02/^=
CF17_F22	4.282 × 10^−01/^=	2.214 × 10^−10/^−	2.800 × 10^−11/^−	7.517 × 10^−03/^−	3.928 × 10^−10/^−	7.321 × 10^−08/^−	4.450 × 10^−08/^−	3.941 × 10^−09/^−	2.800 × 10^−11/^−
CF17_F23	7.389 × 10^−11/^−	3.020 × 10^−11/^−	3.020 × 10^−11/^−	1.031 × 10^−02/^−	8.153 × 10^−11/^−	1.429 × 10^−08/^−	4.943 × 10^−05/^−	4.113 × 10^−07/^−	3.020 × 10^−11/^−
CF17_F24	9.489 × 10^−08/^−	8.367 × 10^−10/^−	2.521 × 10^−11/^−	3.317 × 10^−02/^−	3.936 × 10^−10/^−	1.822 × 10^−06/^−	1.281 × 10^−01/^=	1.618 × 10^−05/^−	3.372 × 10^−03/^−
CF17_F25	1.486 × 10^−04/^−	6.238 × 10^−08/^−	4.470 × 10^−08/^−	3.118 × 10^−05/^−	1.118 × 10^−03/^−	1.605 × 10^−03/^−	1.074 × 10^−04/^−	3.673 × 10^−07/^−	5.741 × 10^−08/^−
CF17_F26	5.029 × 10^−02/^=	4.045 × 10^−06/^−	1.122 × 10^−10/^−	8.466 × 10^−06/^−	4.718 × 10^−09/^−	1.001 × 10^−07/^−	2.383 × 10^−03/^−	1.188 × 10^−07/^−	3.253 × 10^−07/^−
CF17_F27	2.852 × 10^−11/^−	1.020 × 10^−09/^−	2.852 × 10^−11/^−	9.378 × 10^−03/^−	2.852 × 10^−11/^−	1.421 × 10^−07/^−	5.309 × 10^−07/^−	6.283 × 10^−08/^−	3.154 × 10^−11/^−
CF17_F28	2.527 × 10^−10/^−	4.392 × 10^−04/^−	3.343 × 10^−06/^−	5.052 × 10^−02/^=	6.388 × 10^−04/^−	2.820 × 10^−03/^−	2.153 × 10^−02/^−	2.436 × 10^−04/^−	4.426 × 10^−03/^−
CF17_F29	7.389 × 10^−11/^−	4.077 × 10^−11/^−	3.020 × 10^−11/^−	5.072 × 10^−10/^−	3.497 × 10^−09/^−	4.311 × 10^−08/^−	3.646 × 10^−08/^−	5.092 × 10^−08/^−	8.993 × 10^−11/^−
CF17_F30	3.020 × 10^−11/^−	3.020 × 10^−11/^−	3.020 × 10^−11/^−	3.020 × 10^−11/^−	3.010 × 10^−11/^−	3.020 × 10^−11/^−	3.012 × 10^−11/^−	3.020 × 10^−11/^−	3.012 × 10^−11/^−
+/−/=	0/27/2	0/29/0	0/29/0	0/28/1	0/29/0	0/29/0	0/28/1	0/29/0	0/28/1

**Table 6:** Comparison of algorithms in multi-threshold image-segmentation problems.

Algorithms	Time	Parameters
HEOA	2024	$w=0.2\cdot \cos(\frac{\pi }{2}(1-\frac{t}{Max_{iter}}))$
QAGO [52]	2024	No Parameters
IPOA [53]	2024	$F_{PK}=0.4,\;F_{GK}=0.6$
MCOA [54]	2024	$C_{2}=2-(FEs/MaxFEs)$
IMODE [55]	2020	$NP_{init}=18\cdot D,\;NP_{min}=4,\;|A|=2.6\cdot NP,\;p=0.11,\;H=6$

**Table 7:** Algorithm’s fitness function values for multi-threshold image-segmentation problems.

Image	TH	CLNBHEOA		HEOA		QAGO		IPOA		MCOA		IMODE	
		Mean	Std	Mean	Std	Mean	Std	Mean	Std	Mean	Std	Mean	Std
Hunter	2	**2.99 × 10^+03^**	9.33 × 10^−13^	2.98 × 10^+03^	4.40 × 10^+01^	2.98 × 10^+03^	4.53 × 10^−02^	2.96 × 10^+03^	3.35 × 10^+01^	2.98 × 10^+03^	2.89 × 10^+01^	2.96 × 10^+03^	2.18 × 10^+01^
	4	**3.19 × 10^+03^**	8.47 × 10^−02^	3.18 × 10^+03^	2.25 × 10^+01^	3.18 × 10^+03^	3.65 × 10^−01^	3.14 × 10^+03^	2.00 × 10^+01^	**3.19 × 10^+03^**	5.62 × 10^+00^	3.15 × 10^+03^	2.25 × 10^+01^
	6	**3.25 × 10^+03^**	1.37 × 10^−01^	3.24 × 10^+03^	1.16 × 10^+01^	3.24 × 10^+03^	6.26 × 10^−01^	3.20 × 10^+03^	2.61 × 10^+01^	3.24 × 10^+03^	5.08 × 10^+00^	3.20 × 10^+03^	2.17 × 10^+01^
	8	**3.27 × 10^+03^**	4.47 × 10^−01^	**3.27 × 10^+03^**	1.20 × 10^+01^	3.26 × 10^+03^	9.25 × 10^−01^	3.23 × 10^+03^	1.45 × 10^+01^	**3.27 × 10^+03^**	3.50 × 10^+00^	3.23 × 10^+03^	1.55 × 10^+01^
Baboon	2	**1.24 × 10^+03^**	4.67 × 10^−13^	**1.24 × 10^+03^**	0.00 × 10^+00^	1.23 × 10^+03^	7.31 × 10^−02^	1.21 × 10^+03^	3.78 × 10^+01^	1.23 × 10^+03^	3.94 × 10^+01^	1.20 × 10^+03^	2.97 × 10^+01^
	4	**1.38 × 10^+03^**	4.75 × 10^−02^	1.35 × 10^+03^	5.07 × 10^+01^	1.37 × 10^+03^	2.07 × 10^−01^	1.33 × 10^+03^	2.10 × 10^+01^	1.37 × 10^+03^	8.20 × 10^+00^	1.32 × 10^+03^	2.05 × 10^+01^
	6	**1.41 × 10^+03^**	2.60 × 10^−01^	**1.41 × 10^+03^**	9.75 × 10^+00^	1.40 × 10^+03^	8.95 × 10^−01^	1.37 × 10^+03^	1.87 × 10^+01^	**1.41 × 10^+03^**	3.94 × 10^+00^	1.37 × 10^+03^	2.28 × 10^+01^
	8	**1.43 × 10^+03^**	6.94 × 10^−01^	1.42 × 10^+03^	1.99 × 10^+01^	1.42 × 10^+03^	5.54 × 10^−01^	1.39 × 10^+03^	1.90 × 10^+01^	**1.43 × 10^+03^**	1.97 × 10^+00^	1.39 × 10^+03^	1.91 × 10^+01^
Barbara	2	**1.73 × 10^+03^**	0.00 × 10^+00^	1.72 × 10^+03^	3.60 × 10^+01^	1.71 × 10^+03^	1.59 × 10^−01^	1.70 × 10^+03^	2.76 × 10^+01^	1.72 × 10^+03^	3.64 × 10^+01^	1.68 × 10^+03^	6.17 × 10^+01^
	4	**1.92 × 10^+03^**	5.98 × 10^−02^	1.89 × 10^+03^	6.06 × 10^+01^	1.90 × 10^+03^	3.19 × 10^−01^	1.85 × 10^+03^	3.43 × 10^+01^	1.91 × 10^+03^	4.92 × 10^+00^	1.84 × 10^+03^	3.21 × 10^+01^
	6	**1.97 × 10^+03^**	2.89 × 10^−01^	1.96 × 10^+03^	2.83 × 10^+01^	1.93 × 10^+03^	6.42 × 10^−01^	1.91 × 10^+03^	1.89 × 10^+01^	1.96 × 10^+03^	6.28 × 10^+00^	1.91 × 10^+03^	2.54 × 10^+01^
	8	**1.99 × 10^+03^**	4.82 × 10^−01^	1.97 × 10^+03^	3.24 × 10^+01^	1.98 × 10^+03^	5.53 × 10^−01^	1.94 × 10^+03^	1.94 × 10^+01^	**1.99 × 10^+03^**	1.72 × 10^+00^	1.95 × 10^+03^	9.04 × 10^+00^
Camera	2	**5.12 × 10^+03^**	9.33 × 10^−13^	5.11 × 10^+03^	1.87 × 10^−12^	**5.12 × 10^+03^**	5.30 × 10^−02^	5.09 × 10^+03^	3.16 × 10^+01^	5.11 × 10^+03^	1.01 × 10^+01^	5.10 × 10^+03^	1.58 × 10^+01^
	4	**5.24 × 10^+03^**	4.33 × 10^−02^	5.21 × 10^+03^	8.48 × 10^+01^	5.22 × 10^+03^	2.02 × 10^−01^	5.21 × 10^+03^	1.51 × 10^+01^	5.23 × 10^+03^	8.25 × 10^+00^	5.21 × 10^+03^	1.55 × 10^+01^
	6	**5.27 × 10^+03^**	1.39 × 10^+00^	5.26 × 10^+03^	1.90 × 10^+01^	**5.27 × 10^+03^**	1.51 × 10^+00^	5.25 × 10^+03^	7.35 × 10^+00^	**5.27 × 10^+03^**	2.75 × 10^+00^	5.25 × 10^+03^	9.55 × 10^+00^
	8	**5.30 × 10^+03^**	9.68 × 10^−01^	5.03 × 10^+03^	1.18 × 10^+03^	5.28 × 10^+03^	5.94 × 10^−01^	5.27 × 10^+03^	6.27 × 10^+00^	5.29 × 10^+03^	2.92 × 10^+00^	5.27 × 10^+03^	6.27 × 10^+00^
Lena	2	**1.96 × 10^+03^**	7.00 × 10^−13^	1.95 × 10^+03^	2.33 × 10^−13^	1.94 × 10^+03^	2.72 × 10^−01^	1.93 × 10^+03^	2.60 × 10^+01^	1.93 × 10^+03^	1.14 × 10^+01^	1.93 × 10^+03^	2.80 × 10^+01^
	4	**2.19 × 10^+03^**	6.48 × 10^−02^	2.16 × 10^+03^	4.96 × 10^+01^	2.16 × 10^+03^	1.69 × 10^−01^	2.12 × 10^+03^	2.85 × 10^+01^	2.18 × 10^+03^	1.57 × 10^+01^	2.13 × 10^+03^	2.54 × 10^+01^
	6	**2.24 × 10^+03^**	2.78 × 10^−01^	2.22 × 10^+03^	2.60 × 10^+01^	2.23 × 10^+03^	5.20 × 10^−01^	2.19 × 10^+03^	1.92 × 10^+01^	2.23 × 10^+03^	6.37 × 10^+00^	2.18 × 10^+03^	1.61 × 10^+01^
	8	**2.25 × 10^+03^**	3.79 × 10^−01^	2.24 × 10^+03^	1.34 × 10^+01^	2.24 × 10^+03^	7.71 × 10^−01^	2.21 × 10^+03^	1.08 × 10^+01^	2.23 × 10^+03^	2.38 × 10^+00^	2.21 × 10^+03^	1.28 × 10^+01^
Woman	2	**1.48 × 10^+03^**	7.00 × 10^−13^	1.47 × 10^+03^	2.48 × 10^+01^	1.47 × 10^+03^	1.84 × 10^−01^	1.44 × 10^+03^	2.52 × 10^+01^	1.47 × 10^+03^	5.35 × 10^+00^	1.44 × 10^+03^	3.17 × 10^+01^
	4	**1.61 × 10^+03^**	4.75 × 10^−02^	1.60 × 10^+03^	1.97 × 10^+01^	1.60 × 10^+03^	1.58 × 10^−01^	1.56 × 10^+03^	2.55 × 10^+01^	1.60 × 10^+03^	8.50 × 10^+00^	1.57 × 10^+03^	1.88 × 10^+01^
	6	**1.66 × 10^+03^**	7.36 × 10^−01^	1.65 × 10^+03^	7.45 × 10^+00^	1.63 × 10^+03^	7.27 × 10^−01^	1.61 × 10^+03^	2.51 × 10^+01^	1.65 × 10^+03^	5.04 × 10^+00^	1.61 × 10^+03^	2.19 × 10^+01^
	8	**1.67 × 10^+03^**	4.95 × 10^−01^	1.61 × 10^+03^	1.35 × 10^+01^	1.63 × 10^+03^	7.07 × 10^−01^	1.64 × 10^+03^	1.03 × 10^+01^	1.62 × 10^+03^	3.61 × 10^+00^	1.63 × 10^+03^	2.08 × 10^+01^
Friedman Rank		**1.62**		2.88		2.25		5.35		3.49		5.42	
Final Rank		**1.00**		3.00		2.00		5.00		4.00		6.00	

**Table 8:** Algorithm’s PSNR values for multi-threshold image-segmentation problems.

Image	TH	CLNBHEOA		HEOA		QAGO		IPOA		MCOA		IMODE	
		Mean	Std	Mean	Std	Mean	Std	Mean	Std	Mean	Std	Mean	Std
Hunter	2	**1.79 × 10^+01^**	0.00 × 10^+00^	1.78 × 10^+01^	4.35 × 10^−01^	**1.79 × 10^+01^**	7.95 × 10^−03^	1.72 × 10^+01^	1.31 × 10^+00^	**1.79 × 10^+01^**	3.17 × 10^−01^	1.76 × 10^+01^	4.10 × 10^−01^
	4	**2.22 × 10^+01^**	3.14 × 10^−02^	2.20 × 10^+01^	6.15 × 10^−01^	2.21 × 10^+01^	5.00 × 10^−02^	2.00 × 10^+01^	2.76 × 10^+00^	2.20 × 10^+01^	2.80 × 10^−01^	2.07 × 10^+01^	7.56 × 10^−01^
	6	**2.51 × 10^+01^**	3.04 × 10^−02^	2.46 × 10^+01^	7.25 × 10^−01^	2.50 × 10^+01^	2.37 × 10^−01^	2.06 × 10^+01^	3.65 × 10^+00^	2.45 × 10^+01^	4.44 × 10^−01^	2.26 × 10^+01^	9.21 × 10^−01^
	8	**2.68 × 10^+01^**	9.58 × 10^−02^	2.64 × 10^+01^	7.74 × 10^−01^	2.67 × 10^+01^	1.42 × 10^−01^	2.37 × 10^+01^	1.17 × 10^+00^	2.64 × 10^+01^	2.6 × 10^−01^	2.37 × 10^+01^	9.70 × 10^−01^
Baboon	2	**1.53 × 10^+01^**	5.47 × 10^−15^	1.52 × 10^+01^	0.00 × 10^+00^	1.52 × 10^+01^	4.07 × 10^−02^	1.47 × 10^+01^	1.30 × 10^+00^	1.52 × 10^+01^	4.47 × 10^−01^	1.49 × 10^+01^	1.22 × 10^+00^
	4	**2.05 × 10^+01^**	4.10 × 10^−02^	1.98 × 10^+01^	1.86 × 10^+00^	2.04 × 10^+01^	9.46 × 10^−02^	1.80 × 10^+01^	3.10 × 10^+00^	2.02 × 10^+01^	7.08 × 10^−01^	1.84 × 10^+01^	1.54 × 10^+00^
	6	**2.37 × 10^+01^**	1.78 × 10^−01^	2.35 × 10^+01^	1.11 × 10^+00^	2.34 × 10^+01^	3.54 × 10^−01^	2.00 × 10^+01^	3.18 × 10^+00^	2.35 × 10^+01^	6.14 × 10^−01^	2.12 × 10^+01^	1.74 × 10^+00^
	8	**2.63 × 10^+01^**	4.80 × 10^−01^	2.54 × 10^+01^	2.03 × 10^+00^	2.61 × 10^+01^	2.96 × 10^−01^	2.07 × 10^+01^	4.04 × 10^+00^	2.62 × 10^+01^	3.44 × 10^−01^	2.25 × 10^+01^	1.74 × 10^+00^
Barbara	2	**1.64 × 10^+01^**	1.88 × 10^−02^	1.63 × 10^+01^	5.67 × 10^−01^	1.63 × 10^+01^	3.65 × 10^−15^	1.54 × 10^+01^	1.86 × 10^+00^	1.62 × 10^+01^	6.63 × 10^−01^	1.56 × 10^+01^	7.99 × 10^−01^
	4	**1.98 × 10^+01^**	2.33 × 10^−01^	1.92 × 10^+01^	1.35 × 10^+00^	1.96 × 10^+01^	9.48 × 10^−02^	1.87 × 10^+01^	1.00 × 10^+00^	1.97 × 10^+01^	3.52 × 10^−02^	1.86 × 10^+01^	1.19 × 10^+00^
	6	**2.24 × 10^+01^**	3.20 × 10^−01^	2.21 × 10^+01^	1.07 × 10^+00^	2.23 × 10^+01^	1.84 × 10^−01^	1.99 × 10^+01^	1.84 × 10^+00^	2.23 × 10^+01^	1.24 × 10^−01^	2.06 × 10^+01^	1.05 × 10^+00^
	8	**2.43 × 10^+01^**	2.23 × 10^−01^	2.33 × 10^+01^	1.73 × 10^+00^	2.41 × 10^+01^	1.45 × 10^−01^	2.19 × 10^+01^	1.55 × 10^+00^	2.42 × 10^+01^	2.17 × 10^−01^	2.24 × 10^+01^	1.27 × 10^+00^
Camera	2	**1.54 × 10^+01^**	1.08 × 10^+00^	1.49 × 10^+01^	0.00 × 10^+00^	1.50 × 10^+01^	3.67 × 10^−02^	1.50 × 10^+01^	0.00 × 10^+00^	1.50 × 10^+01^	5.03 × 10^−01^	1.49 × 10^+01^	7.56 × 10^−01^
	4	**2.05 × 10^+01^**	6.69 × 10^−02^	2.00 × 10^+01^	1.28 × 10^+00^	2.01 × 10^+01^	8.26 × 10^−02^	1.83 × 10^+01^	2.48 × 10^+00^	2.02 × 10^+01^	7.56 × 10^−01^	1.95 × 10^+01^	1.10 × 10^+00^
	6	**2.34 × 10^+01^**	2.33 × 10^−01^	2.21 × 10^+01^	1.86 × 10^+00^	2.31 × 10^+01^	3.52 × 10^−01^	2.10 × 10^+01^	1.95 × 10^+00^	2.27 × 10^+01^	4.35 × 10^−01^	2.10 × 10^+01^	1.09 × 10^+00^
	8	**2.57 × 10^+01^**	3.57 × 10^−01^	2.47 × 10^+01^	1.55 × 10^+00^	2.54 × 10^+01^	4.20 × 10^−01^	2.20 × 10^+01^	1.75 × 10^+00^	2.50 × 10^+01^	6.05 × 10^−01^	2.27 × 10^+01^	1.00 × 10^+00^
Lena	2	**1.54 × 10^+01^**	1.82 × 10^−15^	1.53 × 10^+01^	1.82 × 10^−15^	1.53 × 10^+01^	2.61 × 10^−02^	1.41 × 10^+01^	1.59 × 10^+00^	1.52 × 10^+01^	4.67 × 10^−01^	1.44 × 10^+01^	8.25 × 10^−01^
	4	**1.86 × 10^+01^**	2.55 × 10^−02^	1.81 × 10^+01^	1.11 × 10^+00^	1.87 × 10^+01^	2.98 × 10^−02^	1.79 × 10^+01^	1.13 × 10^+00^	1.85 × 10^+01^	3.42 × 10^−01^	1.78 × 10^+01^	8.79 × 10^−01^
	6	**2.08 × 10^+01^**	1.70 × 10^−01^	2.05 × 10^+01^	8.75 × 10^−01^	2.07 × 10^+01^	9.41 × 10^−02^	1.97 × 10^+01^	1.68 × 10^+00^	2.07 × 10^+01^	6.10 × 10^−01^	2.01 × 10^+01^	1.22 × 10^+00^
	8	**2.33 × 10^+01^**	5.92 × 10^−01^	2.28 × 10^+01^	1.62 × 10^+00^	2.27 × 10^+01^	8.34 × 10^−01^	1.99 × 10^+01^	3.11 × 10^+00^	2.30 × 10^+01^	8.21 × 10^−01^	2.17 × 10^+01^	1.11 × 10^+00^
Woman	2	**1.47 × 10^+01^**	8.75 × 10^−01^	1.45 × 10^+01^	3.53 × 10^−01^	1.46 × 10^+01^	4.33 × 10^−02^	1.45 × 10^+01^	5.49 × 10^−01^	1.45 × 10^+01^	1.67 × 10^−01^	1.46 × 10^+01^	0.00 × 10^+00^
	4	**2.16 × 10^+01^**	7.27 × 10^−02^	2.11 × 10^+01^	9.23 × 10^−01^	2.16 × 10^+01^	3.69 × 10^−02^	1.86 × 10^+01^	1.83 × 10^+00^	2.14 × 10^+01^	5.02 × 10^−01^	1.96 × 10^+01^	1.56 × 10^+00^
	6	2.39 × 10^+01^	1.15 × 10^−01^	2.33 × 10^+01^	7.01 × 10^−01^	2.37 × 10^+01^	3.31 × 10^−01^	2.05 × 10^+01^	2.40 × 10^+00^	**2.40 × 10^+01^**	7.79 × 10^−01^	2.10 × 10^+01^	1.61 × 10^+00^
	8	**2.71 × 10^+01^**	1.15 × 10^−01^	2.64 × 10^+01^	1.34 × 10^+00^	2.70 × 10^+01^	1.31 × 10^−01^	2.14 × 10^+01^	3.48 × 10^+00^	2.66 × 10^+01^	5.06 × 10^−01^	2.31 × 10^+01^	1.68 × 10^+00^
Friedman Rank		**2.21**		3.22		2.92		4.89		3.01		4.75	
Final Rank		**1**		4		2		6		3		5	

**Table 9:** Algorithm’s SSIM values for multi-threshold image-segmentation problems.

Image	TH	CLNBHEOA		HEOA		QAGO		IPOA		MCOA		IMODE	
		Mean	Std	Mean	Std	Mean	Std	Mean	Std	Mean	Std	Mean	Std
Hunter	2	**7.89 × 10^−01^**	5.37 × 10^−03^	7.61 × 10^−01^	4.39 × 10^−03^	7.61 × 10^−01^	5.64 × 10^−03^	7.58 × 10^−01^	5.86 × 10^−03^	7.60 × 10^−01^	6.18 × 10^−03^	7.63 × 10^−01^	5.09 × 10^−03^
	4	**8.31 × 10^−01^**	6.04 × 10^−03^	8.05 × 10^−01^	2.18 × 10^−03^	8.03 × 10^−01^	2.21 × 10^−03^	8.06 × 10^−01^	2.24 × 10^−03^	8.03 × 10^−01^	2.19 × 10^−03^	8.04 × 10^−01^	2.34 × 10^−03^
	6	**8.44 × 10^−01^**	2.51 × 10^−03^	8.26 × 10^−01^	3.09 × 10^−03^	8.25 × 10^−01^	2.52 × 10^−03^	8.26 × 10^−01^	3.33 × 10^−03^	8.26 × 10^−01^	2.75 × 10^−03^	8.25 × 10^−01^	2.77 × 10^−03^
	8	**8.59 × 10^−01^**	6.34 × 10^−03^	8.38 × 10^−01^	6.09 × 10^−03^	8.38 × 10^−01^	5.98 × 10^−03^	8.39 × 10^−01^	6.33 × 10^−03^	8.39 × 10^−01^	6.39 × 10^−03^	8.40 × 10^−01^	5.07 × 10^−03^
Baboon	2	**7.83 × 10^−01^**	8.90 × 10^−03^	7.55 × 10^−01^	2.61 × 10^−03^	7.55 × 10^−01^	2.71 × 10^−03^	7.55 × 10^−01^	2.86 × 10^−03^	7.55 × 10^−01^	2.93 × 10^−03^	7.55 × 10^−01^	2.82 × 10^−03^
	4	**8.22 × 10^−01^**	5.58 × 10^−03^	8.05 × 10^−01^	3.13 × 10^−03^	8.04 × 10^−01^	2.67 × 10^−03^	8.06 × 10^−01^	2.87 × 10^−03^	8.05 × 10^−01^	2.70 × 10^−03^	8.05 × 10^−01^	3.18 × 10^−03^
	6	**8.74 × 10^−01^**	2.20 × 10^−03^	8.65 × 10^−01^	3.03 × 10^−02^	8.69 × 10^−01^	5.15 × 10^−03^	7.45 × 10^−01^	1.32 × 10^−01^	8.65 × 10^−01^	1.31 × 10^−02^	7.99 × 10^−01^	4.76 × 10^−02^
	8	**9.16 × 10^−01^**	5.31 × 10^−03^	8.91 × 10^−01^	4.99 × 10^−02^	9.15 × 10^−01^	2.85 × 10^−03^	7.48 × 10^−01^	1.69 × 10^−01^	9.13 × 10^−01^	5.23 × 10^−03^	8.31 × 10^−01^	4.12 × 10^−02^
Barbara	2	**7.81 × 10^−01^**	5.76 × 10^−03^	7.55 × 10^−01^	3.64 × 10^−03^	7.54 × 10^−01^	2.84 × 10^−03^	7.56 × 10^−01^	3.18 × 10^−03^	7.55 × 10^−01^	2.79 × 10^−03^	7.55 × 10^−01^	3.04 × 10^−03^
	4	**8.22 × 10^−01^**	5.97 × 10^−03^	8.05 × 10^−01^	2.95 × 10^−03^	8.05 × 10^−01^	3.18 × 10^−03^	8.06 × 10^−01^	3.31 × 10^−03^	8.04 × 10^−01^	2.48 × 10^−03^	8.05 × 10^−01^	3.53 × 10^−03^
	6	**8.40 × 10^−01^**	5.05 × 10^−03^	8.25 × 10^−01^	2.79 × 10^−03^	8.26 × 10^−01^	2.77 × 10^−03^	8.19 × 10^−01^	3.62 × 10^−02^	8.25 × 10^−01^	3.23 × 10^−03^	8.25 × 10^−01^	3.26 × 10^−03^
	8	**8.60 × 10^−01^**	5.42 × 10^−03^	8.42 × 10^−01^	6.39 × 10^−03^	8.40 × 10^−01^	6.26 × 10^−03^	8.39 × 10^−01^	5.76 × 10^−03^	8.40 × 10^−01^	6.53 × 10^−03^	8.42 × 10^−01^	5.76 × 10^−03^
Camera	2	**7.95 × 10^−01^**	3.02 × 10^−03^	7.61 × 10^−01^	5.03 × 10^−03^	7.59 × 10^−01^	6.45 × 10^−03^	7.59 × 10^−01^	5.60 × 10^−03^	7.60 × 10^−01^	5.72 × 10^−03^	7.59 × 10^−01^	5.97 × 10^−03^
	4	**8.30 × 10^−01^**	5.98 × 10^−03^	8.11 × 10^−01^	6.26 × 10^−03^	8.08 × 10^−01^	6.20 × 10^−03^	8.09 × 10^−01^	5.62 × 10^−03^	8.08 × 10^−01^	5.98 × 10^−03^	8.10 × 10^−01^	5.83 × 10^−03^
	6	**8.46 × 10^−01^**	2.36 × 10^−03^	8.25 × 10^−01^	2.74 × 10^−03^	8.25 × 10^−01^	3.19 × 10^−03^	8.25 × 10^−01^	2.92 × 10^−03^	8.25 × 10^−01^	3.04 × 10^−03^	8.25 × 10^−01^	3.08 × 10^−03^
	8	**8.59 × 10^−01^**	6.04 × 10^−03^	8.35 × 10^−01^	2.70 × 10^−03^	8.34 × 10^−01^	2.73 × 10^−03^	8.35 × 10^−01^	2.93 × 10^−03^	8.35 × 10^−01^	2.71 × 10^−03^	8.35 × 10^−01^	2.48 × 10^−03^
Lena	2	**7.94 × 10^−01^**	3.23 × 10^−03^	7.90 × 10^−01^	6.11 × 10^−03^	7.92 × 10^−01^	5.92 × 10^−03^	7.78 × 10^−01^	5.68 × 10^−02^	7.91 × 10^−01^	6.12 × 10^−03^	7.89 × 10^−01^	6.46 × 10^−03^
	4	**8.30 × 10^−01^**	7.12 × 10^−03^	8.07 × 10^−01^	6.06 × 10^−03^	8.10 × 10^−01^	6.71 × 10^−03^	8.10 × 10^−01^	5.71 × 10^−03^	8.08 × 10^−01^	5.90 × 10^−03^	8.13 × 10^−01^	5.63 × 10^−03^
	6	**8.44 × 10^−01^**	2.72 × 10^−03^	8.25 × 10^−01^	2.48 × 10^−03^	8.26 × 10^−01^	2.98 × 10^−03^	8.25 × 10^−01^	2.87 × 10^−03^	8.24 × 10^−01^	2.57 × 10^−03^	8.25 × 10^−01^	3.15 × 10^−03^
	8	**8.63 × 10^−01^**	6.11 × 10^−03^	8.41 × 10^−01^	5.48 × 10^−03^	8.41 × 10^−01^	5.62 × 10^−03^	8.39 × 10^−01^	6.16 × 10^−03^	8.41 × 10^−01^	6.57 × 10^−03^	8.40 × 10^−01^	6.39 × 10^−03^
Woman	2	**7.95 × 10^−01^**	2.86 × 10^−03^	7.64 × 10^−01^	8.70 × 10^−03^	7.66 × 10^−01^	9.26 × 10^−03^	7.66 × 10^−01^	8.17 × 10^−03^	7.65 × 10^−01^	8.74 × 10^−03^	7.63 × 10^−01^	9.42 × 10^−03^
	4	**8.31 × 10^−01^**	4.93 × 10^−03^	8.09 × 10^−01^	6.62 × 10^−03^	8.11 × 10^−01^	6.13 × 10^−03^	8.11 × 10^−01^	6.98 × 10^−03^	8.10 × 10^−01^	5.87 × 10^−03^	8.11 × 10^−01^	6.84 × 10^−03^
	6	**8.45 × 10^−01^**	2.78 × 10^−03^	8.24 × 10^−01^	2.97 × 10^−03^	8.25 × 10^−01^	2.98 × 10^−03^	8.24 × 10^−01^	3.17 × 10^−03^	8.25 × 10^−01^	2.60 × 10^−03^	8.26 × 10^−01^	2.96 × 10^−03^
	8	**8.93 × 10^−01^**	3.90 × 10^−03^	8.65 × 10^−01^	3.00 × 10^−03^	8.64 × 10^−01^	2.87 × 10^−03^	8.66 × 10^−01^	3.09 × 10^−03^	8.65 × 10^−01^	3.22 × 10^−03^	8.66 × 10^−01^	3.31 × 10^−03^
Friedman Rank		**1.15**		3.88		3.81		4.06		3.98		4.13	
Final Rank		**1**		3		2		5		4		6	

**Table 10:** Algorithm’s FSIM values for multi-threshold image-segmentation problems.

Image	TH	CLNBHEOA		HEOA		QAGO		IPOA		MCOA		IMODE	
		Mean	Std	Mean	Std	Mean	Std	Mean	Std	Mean	Std	Mean	Std
Hunter	2	**7.96 × 10^−01^**	3.04 × 10^−03^	7.67 × 10^−01^	1.45 × 10^−02^	7.68 × 10^−01^	1.35 × 10^−02^	7.69 × 10^−01^	1.32 × 10^−02^	7.67 × 10^−01^	1.35 × 10^−02^	7.67 × 10^−01^	1.76 × 10^−02^
	4	**8.54 × 10^−01^**	2.28 × 10^−04^	8.46 × 10^−01^	1.98 × 10^−02^	8.52 × 10^−01^	1.71 × 10^−03^	7.89 × 10^−01^	8.94 × 10^−02^	8.49 × 10^−01^	6.68 × 10^−03^	8.13 × 10^−01^	1.76 × 10^−02^
	6	**9.09 × 10^−01^**	3.70 × 10^−04^	9.00 × 10^−01^	1.69 × 10^−02^	9.07 × 10^−01^	1.56 × 10^−03^	8.06 × 10^−01^	9.06 × 10^−02^	9.01 × 10^−01^	6.85 × 10^−03^	8.54 × 10^−01^	2.19 × 10^−02^
	8	**9.39 × 10^−01^**	4.39 × 10^−04^	9.34 × 10^−01^	1.67 × 10^−02^	9.38 × 10^−01^	1.69 × 10^−03^	8.79 × 10^−01^	2.76 × 10^−02^	9.35 × 10^−01^	5.54 × 10^−03^	8.86 × 10^−01^	1.75 × 10^−02^
Baboon	2	**7.95 × 10^−01^**	2.61 × 10^−03^	7.64 × 10^−01^	1.47 × 10^−02^	7.67 × 10^−01^	1.63 × 10^−02^	7.68 × 10^−01^	1.32 × 10^−02^	7.68 × 10^−01^	1.58 × 10^−02^	7.61 × 10^−01^	1.68 × 10^−02^
	4	**8.43 × 10^−01^**	8.85 × 10^−04^	8.16 × 10^−01^	6.17 × 10^−02^	8.42 × 10^−01^	1.75 × 10^−03^	7.69 × 10^−01^	1.11 × 10^−01^	8.35 × 10^−01^	1.82 × 10^−02^	7.82 × 10^−01^	4.18 × 10^−02^
	6	**8.99 × 10^−01^**	2.85 × 10^−03^	8.93 × 10^−01^	2.38 × 10^−02^	8.96 × 10^−01^	3.64 × 10^−03^	8.08 × 10^−01^	1.01 × 10^−01^	8.95 × 10^−01^	1.26 × 10^−02^	8.51 × 10^−01^	3.48 × 10^−02^
	8	**9.34 × 10^−01^**	6.87 × 10^−03^	9.17 × 10^−01^	3.76 × 10^−02^	**9.34 × 10^−01^**	4.81 × 10^−03^	8.09 × 10^−01^	1.29 × 10^−01^	**9.34 × 10^−01^**	5.48 × 10^−03^	8.73 × 10^−01^	3.66 × 10^−02^
Barbara	2	**7.85 × 10^−01^**	2.13 × 10^−03^	7.61 × 10^−01^	7.18 × 10^−03^	7.66 × 10^−01^	8.16 × 10^−03^	7.64 × 10^−01^	8.73 × 10^−03^	7.65 × 10^−01^	1.48 × 10^−02^	7.66 × 10^−01^	8.71 × 10^−03^
	4	**8.07 × 10^−01^**	6.96 × 10^−04^	7.89 × 10^−01^	3.32 × 10^−02^	**8.07 × 10^−01^**	6.93 × 10^−04^	7.74 × 10^−01^	2.36 × 10^−02^	8.06 × 10^−01^	2.64 × 10^−03^	7.68 × 10^−01^	2.75 × 10^−02^
	6	**8.63 × 10^−01^**	1.41 × 10^−03^	8.56 × 10^−01^	2.38 × 10^−02^	8.62 × 10^−01^	1.11 × 10^−03^	7.85 × 10^−01^	4.40 × 10^−02^	8.58 × 10^−01^	7.46 × 10^−03^	8.05 × 10^−01^	1.97 × 10^−02^
	8	**8.93 × 10^−01^**	7.43 × 10^−04^	8.67 × 10^−01^	3.89 × 10^−02^	8.92 × 10^−01^	1.08 × 10^−03^	8.28 × 10^−01^	3.12 × 10^−02^	8.92 × 10^−01^	2.84 × 10^−03^	8.47 × 10^−01^	1.49 × 10^−02^
Camera	2	**7.85 × 10^−01^**	3.06 × 10^−03^	7.65 × 10^−01^	8.23 × 10^−03^	7.69 × 10^−01^	8.25 × 10^−03^	7.64 × 10^−01^	7.83 × 10^−03^	7.64 × 10^−01^	9.72 × 10^−03^	7.60 × 10^−01^	8.44 × 10^−03^
	4	**8.28 × 10^−01^**	1.20 × 10^−03^	8.17 × 10^−01^	2.81 × 10^−02^	8.27 × 10^−01^	1.09 × 10^−03^	7.93 × 10^−01^	3.17 × 10^−02^	8.18 × 10^−01^	2.07 × 10^−02^	7.93 × 10^−01^	2.08 × 10^−02^
	6	**8.74 × 10^−01^**	1.33 × 10^−02^	8.46 × 10^−01^	3.89 × 10^−02^	8.73 × 10^−01^	7.82 × 10^−03^	8.33 × 10^−01^	4.48 × 10^−02^	8.66 × 10^−01^	1.53 × 10^−02^	8.31 × 10^−01^	2.48 × 10^−02^
	8	**9.13 × 10^−01^**	3.20 × 10^−03^	9.01 × 10^−01^	2.20 × 10^−02^	9.12 × 10^−01^	3.48 × 10^−03^	8.57 × 10^−01^	2.12 × 10^−02^	9.03 × 10^−01^	1.02 × 10^−02^	8.74 × 10^−01^	1.69 × 10^−02^
Lena	2	**7.91 × 10^−01^**	5.97 × 10^−03^	7.64 × 10^−01^	8.86 × 10^−03^	7.60 × 10^−01^	7.93 × 10^−03^	7.65 × 10^−01^	7.98 × 10^−03^	7.64 × 10^−01^	8.87 × 10^−03^	7.65 × 10^−01^	8.40 × 10^−03^
	4	**8.28 × 10^−01^**	5.54 × 10^−03^	8.13 × 10^−01^	5.33 × 10^−03^	8.10 × 10^−01^	6.83 × 10^−03^	8.08 × 10^−01^	5.34 × 10^−03^	8.08 × 10^−01^	5.53 × 10^−03^	8.10 × 10^−01^	4.89 × 10^−03^
	6	**8.53 × 10^−01^**	8.15 × 10^−04^	8.42 × 10^−01^	2.33 × 10^−02^	8.52 × 10^−01^	8.29 × 10^−04^	7.99 × 10^−01^	3.39 × 10^−02^	8.44 × 10^−01^	1.05 × 10^−02^	8.02 × 10^−01^	2.02 × 10^−02^
	8	**8.71 × 10^−01^**	2.33 × 10^−03^	8.63 × 10^−01^	2.08 × 10^−02^	8.70 × 10^−01^	1.50 × 10^−03^	8.02 × 10^−01^	5.88 × 10^−02^	8.67 × 10^−01^	3.98 × 10^−03^	8.34 × 10^−01^	1.62 × 10^−02^
Woman	2	**7.94 × 10^−01^**	2.56 × 10^−03^	7.75 × 10^−01^	2.92 × 10^−03^	7.74 × 10^−01^	2.58 × 10^−03^	7.75 × 10^−01^	2.72 × 10^−03^	7.76 × 10^−01^	2.66 × 10^−03^	7.74 × 10^−01^	2.57 × 10^−03^
	4	**8.16 × 10^−01^**	7.67 × 10^−04^	8.05 × 10^−01^	2.28 × 10^−02^	8.15 × 10^−01^	9.54 × 10^−04^	7.50 × 10^−01^	2.51 × 10^−02^	8.08 × 10^−01^	1.30 × 10^−02^	7.86 × 10^−01^	1.79 × 10^−02^
	6	**8.84 × 10^−01^**	1.90 × 10^−03^	8.81 × 10^−01^	1.70 × 10^−02^	8.83 × 10^−01^	2.89 × 10^−03^	7.86 × 10^−01^	5.95 × 10^−02^	8.70 × 10^−01^	1.23 × 10^−02^	8.00 × 10^−01^	3.38 × 10^−02^
	8	**9.17 × 10^−01^**	1.86 × 10^−03^	9.05 × 10^−01^	3.04 × 10^−02^	9.16 × 10^−01^	1.76 × 10^−03^	7.99 × 10^−01^	7.89 × 10^−02^	9.07 × 10^−01^	9.87 × 10^−03^	8.37 × 10^−01^	3.21 × 10^−02^
Friedman Rank		**1.76**		3.05		2.93		4.88		3.43		4.94	
Final Rank		**1**		3		2		5		4		6	

## Data Availability

Data requests can be emailed to the corresponding author’s email address.
